# Linkage disequilibrium mapping for grain Fe and Zn enhancing QTLs useful for nutrient dense rice breeding

**DOI:** 10.1186/s12870-020-2262-4

**Published:** 2020-02-04

**Authors:** S. K. Pradhan, E. Pandit, S. Pawar, R. Naveenkumar, S. R. Barik, S. P. Mohanty, D. K. Nayak, S. K. Ghritlahre, D. Sanjiba Rao, J. N. Reddy, S. S. C. Patnaik

**Affiliations:** 1 ICAR-National Rice Research Institute, Cuttack, Odisha India; 2 ICAR-Indian Institute of Rice Research, Hyderabad, India

**Keywords:** Association study, Linkage disequilibrium, Grain Fe content, Grain Zn content, Biofortification

## Abstract

**Background:**

High yielding rice varieties are usually low in grain iron (Fe) and zinc (Zn) content. These two micronutrients are involved in many enzymatic activities, lack of which cause many disorders in human body. Bio-fortification is a cheaper and easier way to improve the content of these nutrients in rice grain.

**Results:**

A population panel was prepared representing all the phenotypic classes for grain Fe-Zn content from 485 germplasm lines. The panel was studied for genetic diversity, population structure and association mapping of grain Fe-Zn content in the milled rice. The population showed linkage disequilibrium showing deviation of Hardy-Weinberg’s expectation for Fe-Zn content in rice. Population structure at K = 3 categorized the panel population into distinct sub-populations corroborating with their grain Fe-Zn content. STRUCTURE analysis revealed a common primary ancestor for each sub-population. Novel quantitative trait loci (QTLs) namely *qFe3.3* and *qFe7.3* for grain Fe and *qZn2.2*, *qZn8.3* and *qZn12.3* for Zn content were detected using association mapping. Four QTLs, namely *qFe3.3*, *qFe7.3*, *qFe8.1* and *qFe12.2* for grain Fe content were detected to be co-localized with *qZn3.1*, *qZn7*, *qZn8.3* and *qZn12.3* QTLs controlling grain Zn content, respectively. Additionally, some Fe-Zn controlling QTLs were co-localized with the yield component QTLs, *qTBGW*, *OsSPL14* and *qPN*. The QTLs *qFe1.1*, *qFe3.1*, *qFe5.1, qFe7.1, qFe8.1, qZn6, qZn7 and gRMm9–1* for grain Fe-Zn content reported in earlier studies were validated in this study.

**Conclusion:**

Novel QTLs, *qFe3.3* and *qFe7.3* for grain Fe and *qZn2.2*, *qZn8.3* and *qZn12.3* for Zn content were detected for these two traits. Four Fe-Zn controlling QTLs and few yield component QTLs were detected to be co-localized. The QTLs, *qFe1.1*, *qFe3.1*, *qFe5.1, qFe7.1, qFe8.1, qFe3.3, qFe7.3, qZn6, qZn7, qZn2.2, qZn8.3* and *qZn12.3* will be useful for biofortification of the micronutrients. Simultaneous enhancement of Fe-Zn content may be possible with yield component traits in rice.

## Background

Majority of the global population consume rice daily, particularly in Asiatic countries. But, rice grain is poor source of micronutrients such as iron (Fe) and zinc (Zn). The practice of consuming polished rice as a staple food in India aggravates malnutrition. Substantial amounts of iron and zinc are removed during milling. The polished rice contains around 2 mg kg^− 1^ Fe while the recommended dietary intake is 10–15 mg kg^− 1^. Similarly, polished rice contains around 12 mg kg^− 1^ of Zn, whereas the recommended intake for humans is 12–15 mg kg^− 1^ [1]. Iron is an important constituent of haemoglobin in red blood cells and is essential for the proper functioning of several enzymes in the body. Zinc is required for the metabolic activity of 300 enzymes, and is essential for enzymes involved in cell division, protein synthesis and growth [2]. To date, proper attention has not been given for improvement of these micronutrients in rice grain. Literature survey on existence of grain Fe and Zn content diversity reported in rice is high in natural rice germplasms [3–13]. Increasing the iron and zinc content in rice grain through breeding is cheaper and an easier option to reduce malnutrition in the developing countries. Increasing Fe and Zn content in rice is possible by utilizing elite germplasms possessing enormous genetic potential for grain Fe-Zn content in rice breeding program [5, 6, 13–15].

Popular rice varieties usually contain lesser micronutrients in grains compared to the traditional cultivars and landraces [16, 17]. QTLs responsible for further enhancing these nutrients in the high yielding rice varieties need to be identified and utilized. Through classical breeding approach, a very limited success could be achieved in this context. Earlier reports on low heritability of micronutritients concentration, negative relationship with high grain yield and genotype and environment interaction were the main limitations of genetic enhancement for these nutrients [18–21]. However, the development of CR Dhan 311, DRR Dhan 45 and Chhattishgarh Zinc Rice-1 are the recent examples of high yielding varieties in India with high grain Zn content. But, these achievements were through non-targeted classical breeding approach. Success in breeding for micronutrients enrichment is limited due to involvement of many quantitative trait loci (QTLs) with small effects and interacts highly with the environmental factors. Precision breeding is a better approach for enriching the elite rice varieties with desired micronutrients. The availability of information on robust markers for different QTLs and potential donors are pre-requisite for the success of precision breeding. Several QTLs for grain Fe and Zn related traits have been reported in rice from different genetic backgrounds of intraspecific and interspecific crosses [6, 9, 13–15, 18–36] are hardly being in use in modern molecular breeding for Fe-Zn enhancement.

Mapping reports of Fe-Zn content in rice indicated the use of various bi-parental mapping populations for detecting the genes controlling these two micronutrients. Gene mapping using bi-parental mapping populations is more time consuming, costlier and lesser resolution than association mapping [17, 37–40]. These limitations can be overcome by linkage disequilibrium (LD) mapping or association mapping. Association mapping is based on linkage disequilibrium or variations existing in natural or developed populations [17, 37, 39, 40]. The main purpose of association mapping here is to estimate the correlations between molecular markers with the grain Fe and Zn content in a panel containing representative rice population that possess considerable amount of variability for these two traits. Only few research reports on association mapping for grain Fe-Zn content are available [41, 42]. But, report on QTL mapping through association mapping in rice are available for grain yield and agronomic traits [29, 41, 43, 44]; seedling stage cold tolerance [40, 45]; cold tolerance at germination and booting stages [46]; high temperature stress tolerance [39]; grain quality traits [47]; salinity tolerance [48]; drought tolerance traits [49, 50]; early seedling vigour [51] and grain protein content [17]. However, the information on association mapping for grain Fe and Zn content in rice is scarce. In this study, a panel population containing 102 genotypes shortlisted from 485 germplasm lines were analyzed using 100 SSR and gene specific molecular markers for genetic diversity, population structure and association mapping for grain Fe and Zn content.

## Results

### Phenotyping of the panel population for grain Fe and Zn content in milled rice

**Fig. 1:**
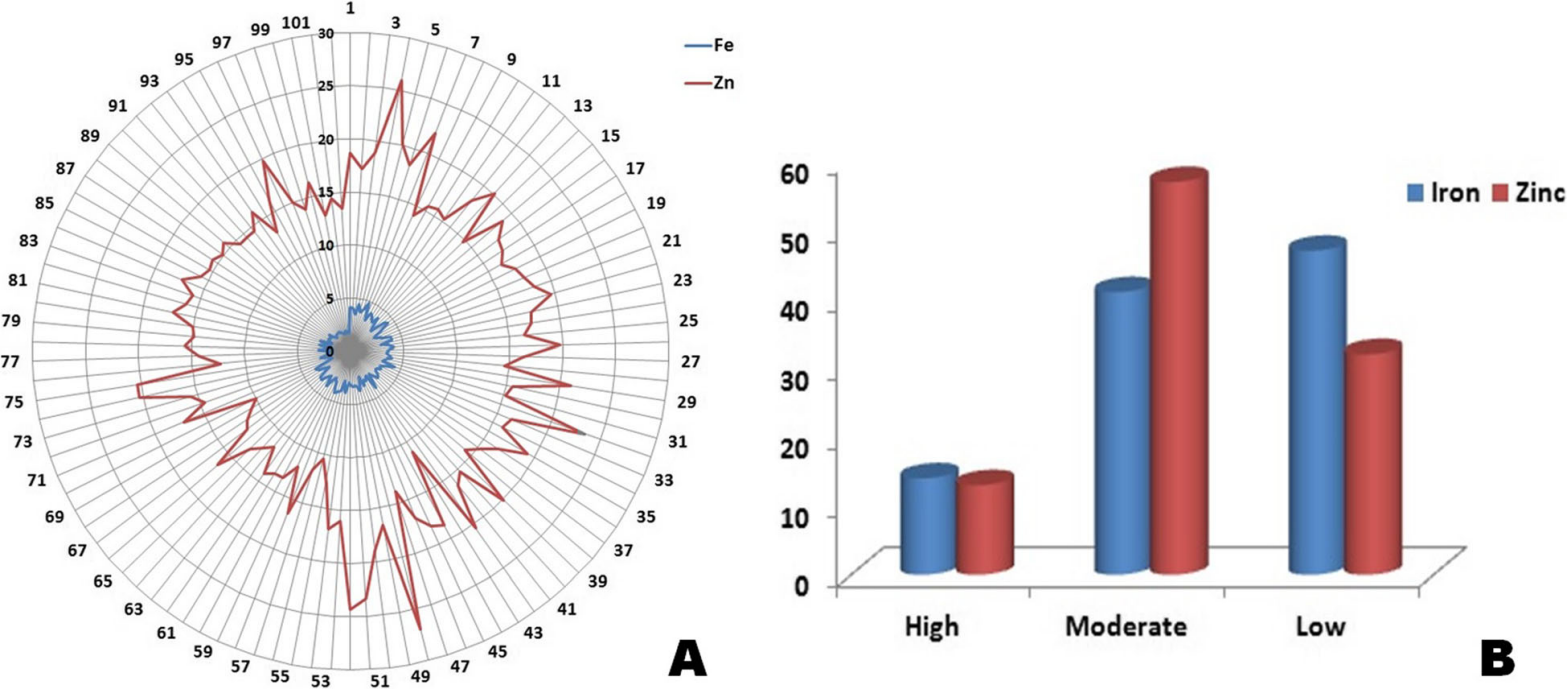
Fe and Zn content of 102 genotypes and their frequency distribution in the panel population. **a** Spider graph showing the Fe and Zn content of the genotypes. **b** Frequency of high, moderate and low Fe-Zn genotypes in the panel population

The panel population was prepared by shortlisting genotypes from different phenotypic groups based on screening results of 485 germplasm lines for Fe-Zn content. Almost equal proportions of genotypes were pooled from each Fe-Zn phenotypic group along with the check varieties for constitution of the panel containing 102 germplasm lines. The mean iron content in of the panel population varied from 1.07 to 5.38 mg kg^− 1^ in the milled rice phenotyping results of wet seasons, 2016 and 2017. A higher Fe-content of > 4 mg kg^− 1^ in milled rice was observed in 13 genotypes viz.*,* Chittimuthylu, IET25450, IET 24775, IET 24760, IET 24316, BPT5204, IET 23832, Kalanamak, IET25441 and IET25465, IET 23829 and IET 24779 (Additional file 3: Table S1). The check varieties for micronutrients viz., Chittimuthylu and Kalanamak showed mean grain Fe content of 4.8 and 4.48 mg kg^− 1^, respectively. Genotypes containing ≥25 mg kg^− 1^ zinc and /or ≥ 10 mg kg^− 1^ iron in brown rice along with at par or higher grain yield compare to the yield checks are considered desirable biofortified rice lines. While comparing in milled rice, genotype containing ≥4 mg kg^− 1^ Fe and ≥ 20 mg kg^− 1^ Zn and producing higher yield than yield check variety is considered as desirable genotypes. It is observed that the genotypes enlisted in the Table containing > 4 mg kg^− 1^ Fe in milled rice were found to be with > 12 mg kg^− 1^ in brown rice. Similarly, genotypes present in the panel with > 20 mg kg^− 1^ Zn content in milled rice showed > 25 mg kg^− 1^ in brown rice. From the results, nine biofortified rice genotypes, two micronutrients check cultivars and landrace Sadakadam showed more than 4 mg kg^− 1^ Fe in milled rice and categorized under high Fe containing genotypes in our study (Table 1). Forty two genotypes comprised of the moderate group (3–4 mg kg^− 1^ Fe in milled rice) while 48 showed low values (< 3 mg kg^− 1^ Fe). Mean zinc content in milled rice varied from 7.43–27.97 mg kg^− 1^ showing ≥20 mg kg^− 1^ in fourteen genotypes (Additional file 3: Table S1). These fourteen genotypes were categorized under high grain Zn containing rice. The two check varieties for micronutrients, Chittimuthylu and Kalanamak had mean value of 22.3 mg kg^− 1^ and 25.91 mg kg^− 1^ zinc in grain, respectively. In the studied panel, 56 genotypes showed moderate level of 15–20 mg kg^− 1^ grain Zn, while 32 lines exhibited low level (< 15 mg kg^− 1^ grain Zn). Evaluation of 102 genotypes for grain yield and Fe-Zn content over 2 years revealed two biofortified lines namely IET24779 and IET25465 showing at par yield with the yield check variety IR64 (Table 1). The genotypes containing high Fe or high Zn in milled rice and at par grain yield with yield check variety may be selected as cultivar or as donor parent for the breeding programs. The desirable genotypes with high Fe content and good grain yield were IET23829, IET 23832, BPT5204, IET24316, IET24760, IET24775, IET24779, IET25441 and IET25465. The genotypes with high Zn content and high grain yield were IET23824, IET24391, IET25446, IET25461, IET25465, IET25457, IET25477, Mumihunger, Sneha, Lalat and Chittimuthyalu. Genotypes IET24779, IET25465 and Chittimuthyalu produced at par yield with standard check variety IR64 along with high grain Fe-Zn content. The frequency of genotypes having low, moderate and high grain Fe and Zn content is depicted in spider graph and histogram (Fig. 1).

**Table 1:** Mean estimate values of days to 50% flowering, grain Fe- Zn content, panicles/m^2^ and grain yield of 102 genotypes including biofortified lines and check varieties studied during wet season, 2016 & 2017

Sl. No.	National testing No./Accession No./Name of the genotype	Days to 50% flowering	Iron content in ppm	Zinc content in ppm	Panicles/m^2^	Grain yield (kg/ha)
1	IET23829	99	4.18	18.73	282	3950
2	IR64	91	3.97	17.24	281	4250
3	IET23834	88	3.5	18.8	264	3450
4	Kalanamak	106	4.48	25.91	265	2962
5	IET23824	90	3.75	20.1	262	3771
6	IET23832	97	4.15	18.43	266	4068
7	Chittimuthyalu	110	4.8	22.03	283	3620
8	IET24780	94	3.21	14.09	295	4552
9	BPT5204	111	4.11	15.46	285	4431
10	IET24771	107	3.9	15.68	286	4958
11	IET24766	94	3.41	15.2	279	4281
12	IET24316	87	4.04	18.25	264	3532
13	IET24391	110	2.99	20.11	249	3984
14	IET24777	96	2.73	14.8	295	4848
15	IET24760	110	4.11	18.88	303	4693
16	IET24775	110	4.33	17.47	284	4623
17	IET24783	96	3.35	17.18	275	4883
18	IET24544	98	2.49	16.4	286	4899
19	IET24336	93	2.77	17.37	269	4598
20	IET24772	96	3.26	17.8	287	4285
21	IET24557	107	3.97	18.34	288	4079
22	IET24779	102	4.02	19.67	280	4283
23	IET24774	104	2.93	17.4	293	4685
24	IET24787	97	3.62	17.2	280	3826
25	IET25441	101	4.06	16.48	275	4258
26	IET25443	109	3.6	19.73	284	3629
27	IET25444	111	3.45	16.31	257	4229
28	IET25445	106	3.57	14.55	287	4399
29	IET25446	94	3.95	21.02	284	3857
30	IET25447	105	3.5	15.57	290	4748
31	IET25449	98	3.19	15.2	266	4379
32	IET25450	99	4.41	23.43	282	3133
33	IET25452	106	3.41	16.42	280	4490
34	IET25453	93	3.03	15.95	269	4515
35	IET25454	87	3.33	19.27	227	2550
36	IET25457	99	2.9	16.71	246	3582
37	IET25459	102	2.97	15.22	272	4479
38	IET25460	105	3.41	14.22	284	5210
39	IET25461	95	3.53	20.14	256	3767
40	IET25463	98	3.09	15.32	276	4843
41	IET25464	94	2.76	16.2	243	3392
42	IET25465	101	4.19	20.36	265	3656
43	Gontra Bidhan3	106	2.7	11.1	251	4050
44	IET25469	95	3.15	18.57	276	4054
45	IET25470	96	2.98	18.1	263	4772
46	IET25471	103	3.44	16.87	265	4276
47	DRRH3	101	2.53	13.88	253	5162
48	IET25472	92	3.78	26.96	275	3059
49	IET25473	103	3.35	16.62	280	5002
50	IET25474	105	3.26	18.95	269	4125
51	IET25475	91	3.37	23.35	267	3881
52	IET25477	101	3.13	24.28	284	3620
53	IET25478	105	2.94	16	271	4209
54	IET25479	106	3.94	16.84	290	4424
55	Lalmeeta	118	2.79	12.66	252	3430
56	Abhimanyu	119	3.78	10.44	272	3810
57	Kalobhutia	121	3.96	11.7	246	3360
58	Sadakajam	115	4.17	16.32	246	3210
59	Geetanjali	128	3.18	11.94	280	3950
60	Kakhru	127	2.85	13.56	254	3740
61	Boanti	120	3.18	13.56	248	3430
62	Tulsimukul	120	3.99	14.1	226	2830
63	Kokilpatri	112	2.94	11.52	252	3280
64	Basmatikarnal	125	3.21	12.3	242	3120
65	Kalonunia	132	3.84	13.2	218	2930
66	SafedLuchai2	108	3.69	16.5	220	2940
67	Bankra	137	2.49	12.24	248	3320
68	Moongi	126	2.85	11.7	198	2830
69	Swarnakranti	124	3.42	10.86	274	3810
70	Kalojeera	99	3.69	9.96	188	2820
71	Ketekijoha	114	2.49	17.04	230	4130
72	Maudamani	109	1.85	14.55	210	7740
73	Tarori Basmati	113	2.01	15.71	218	2860
74	Mamihunger	110	2.92	20.36	175	2640
75	Sneha	112	2.31	20.36	240	3860
76	Savitri	123	2.07	12.32	275	5680
77	CR Dhan 101	94	1.86	14.28	225	4670
78	CR Dhan 907	112	3.05	15.59	280	4250
79	CR Dhan 801	114	1.92	14.84	305	6340
80	Chinikamini	116	2.46	15.04	226	3680
81	Nuakalajeera	112	2.98	17.16	210	3670
82	Moti	113	1.98	16.12	265	4760
83	Nuadhusura	114	3.05	15.82	232	3580
84	Heera	70	1.85	17.23	190	2940
85	Jalmagna	130	2.32	15.76	236	3250
86	AC44756	110	2.35	15.32	180	2240
87	AC44755	112	2.23	15.63	178	2350
88	AC44754	112	2.53	15.08	192	2420
89	AC44753	110	2.13	15.73	184	2480
90	Swarna-Sub 1	116	1.81	14.48	286	6130
91	Ranjit	127	1.96	14.52	308	5620
92	Swarna	120	2.03	14.52	310	6350
93	Jaya	108	2.05	16.08	274	4580
94	Samalei	110	2.1	13.16	262	4450
95	AC44752	112	1.9	16.24	186	2520
96	Lalat	98	1.86	20.05	246	4520
97	MTU1010	97	1.79	15.04	258	4890
98	Naveen	101	1.73	14.08	268	5120
99	Satabdi	91	2.01	16.4	252	4460
100	Pooja	126	1.73	13.07	284	5470
101	Sarala	133	1.92	14.52	278	5120
102	Agnisar	105	1.75	13.46	186	2620
	LSD_5%_	4.551	0.561	3.140	59.01	967
	CV%	2.2	9.3	9.7	11.5	12.0

### Relatedness among genotypes for grain yield and Fe-Zn content through genotype-by-trait biplot analysis

**Fig. 2:**
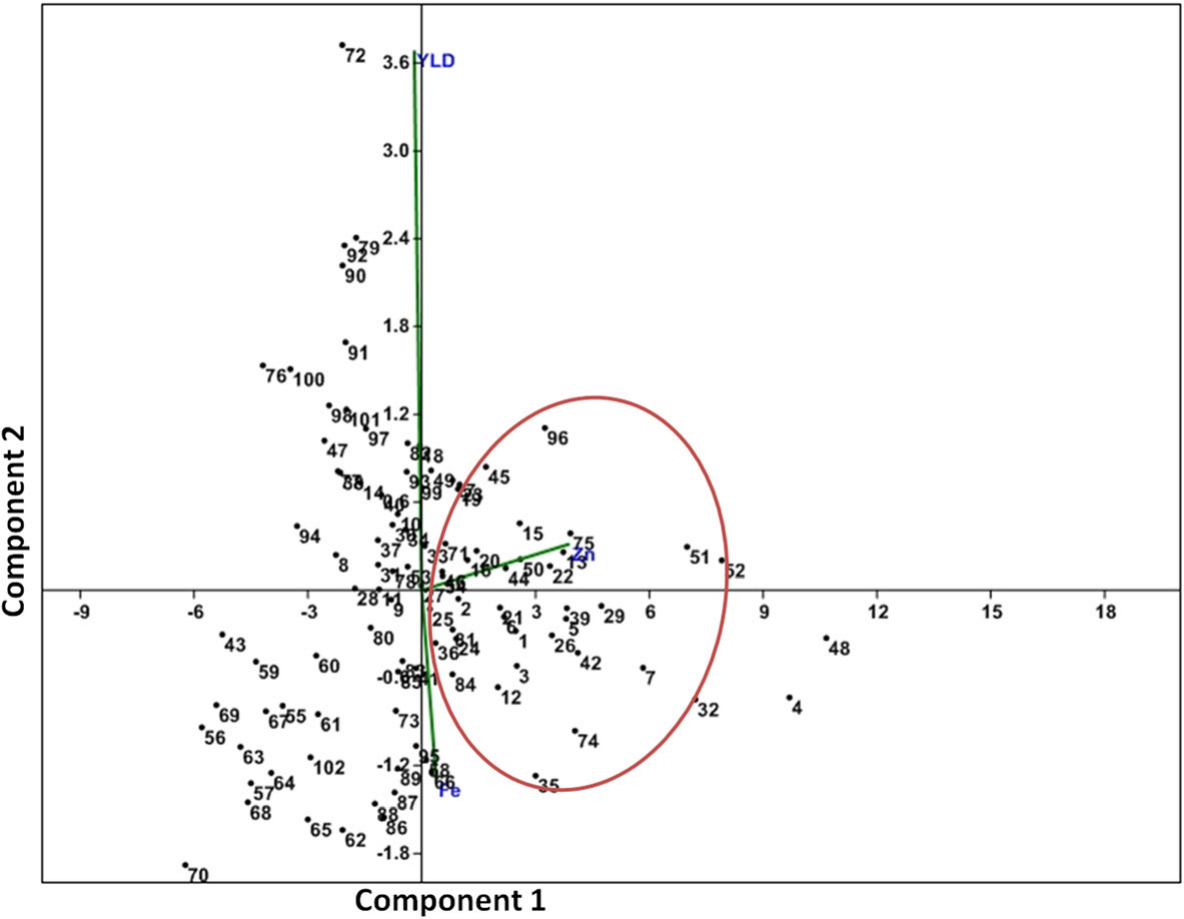
Genotype-by-trait biplot graph showing 102 genotypes in two main principal components for three traits. Fe: grain iron content; Zn: grain zinc content; yld: grain yield (kg/ha); PN: panicle number; DFF: days to 50% flowering. The dot numbers in the figure represent the serial number of the genotypes enlisted in Table 1

The scatter diagram was plotted in the first two principal components axis to generate genotype-by-trait biplot graph for grain Fe, grain Zn concentration and grain yield of the 102 genotypes present in the panel (Fig. 2). The first and second principal components had 87.82 and 8.08% of the total variability with eigen value of 10.18 and 0.937, respectively (Additional file 1: Figure S1). Among the 3 traits from the principal component analysis, the grain yield contributed maximum diversity, followed by grain Fe content and grain Zn content in the panel population (Fig. 2). From the scattering pattern of genotypes in the 4 quadrants revealed the placement of the genotypes for high grain yield and Fe content in opposite direction. Higher Zn containing genotypes are in between grain yield and Fe content. However, there are some genotypes which are located nearer to origin possessing higher estimates of grain yield and grain Fe-Zn content. These genotypes have been encircled in the figure (Fig. 2). The top right (I^st^ quadrant) and bottom right (2nd quadrant) possessed majority of the genotypes with better in Fe and Zn content in kernel along with higher grain yield. The 3rd (bottom left) and 4th quadrant (top left) accommodates genotypes most of which were poor in grain Fe and Zn content with low grain yield (Fig. 2). Many genotypes with moderate in Fe and Zn content are found in 2nd quadrant. The desirable genotypes containing moderate to high grain Fe- Zn and grain yield are located both side of the X-axis and are encircled in the scatter diagram (Fig. 2).

### Genetic diversity in the panel population using 100 molecular markers

The panel containing 102 germplasm lines exhibiting wide genetic variation for grain Fe and Zn content were genotyped using 100 molecular markers including 25 gene specific and 75 SSR markers. The loci used for genetic diversity and the calculated parameters are depicted in the table (Table 2). Two hundred forty four amplicons were obtained in toto with 2.44 average alleles per locus. The number of alleles ranged from 1 to 6 per marker with RM340 showing the highest number of alleles in the panel genotypes. The average major allele frequency of Fe and Zn linked polymorphic markers was observed to be 0.675 ranging within the bracket of 0.4023 (RM152) and 1.0000 (RM309, RM452, RM137, RM1789 and RM441) (Table 2). The PIC value ranged from 0.000 (RM6209, sZIP8, RM3409, RM309, RM452, RM137, RM1789, RM556, RM23, RM34, RM441 and GRMM9–2) to 0.6713 (RM407) with mean PIC of 0.3553. The observed average heterozygosity (Ho) was 0.0496 with a range of 0.00–0.6714. Amongst the markers used, only 36 showed heterogygocity value to be more than zero, whereas 64 exhibited zero values. The average gene diversity (He) varied from 0.0000 (RM309, RM452, RM137, RM1789, RM44, RM590 and RM258) to 0.6711 (RM3392) with an average value of 0.35.

**Table 2:** Details of 100 SSR and direct marker loci used for genotyping a panel containing 102 rice genotypes and their genetic diversity parameters

Sl. No.	Marker Name	No. of alleles	Range of amplicon (bp)	Major allele frequency	Gene diversity	Hetero- zygosity	PIC value	inbreeding coefficient (f)
1	RM243	3.0000	100–140	0.6238	0.5331	0.1980	0.4705	0.6315
2	RM488	2.0000	190–210	0.7344	0.3901	0.0104	0.3140	0.9736
3	RM490	2.0000	95–115	0.6450	0.4580	0.0500	0.3531	0.8919
4	RM574	2.0000	150–160	0.8618	0.2381	0.0132	0.2098	0.9455
5	RM122	2.0000	240–260	0.6480	0.4562	0.1939	0.3521	0.5785
6	RM234	3.0000	150–160	0.6050	0.5407	0.0500	0.4704	0.9084
7	RM248	3.0000	95–115	0.3670	0.6626	0.1383	0.5884	0.7933
8	RM8007	2.0000	155–175	0.5479	0.4954	0.1809	0.3727	0.6381
9	RM17	2.0000	150–170	0.5990	0.4804	0.0521	0.3650	0.8927
10	RM260	2.0000	100–130	0.7407	0.3841	0.0370	0.3103	0.9053
11	RM7	3.0000	140–400	0.4516	0.6374	0.0000	0.5625	1.0000
12	RM517	2.0000	250–270	0.7590	0.3658	0.0000	0.2989	1.0000
13	RM501	2.0000	150–165	0.7611	0.3636	0.0556	0.2975	0.8488
14	OsZIP4	2.0000	310–310	0.6569	0.4508	0.0000	0.3492	1.0000
15	RM594	2.0000	295–310	0.8110	0.3066	0.1585	0.2596	0.4876
16	RM3412	3.0000	200–260	0.5506	0.5936	0.0225	0.5261	0.9626
17	RM5638	2.0000	200–250	0.5956	0.4817	0.0735	0.3657	0.8494
18	RM6712	3.0000	105–200	0.6071	0.5541	0.3878	0.4941	0.3049
19	RM168	2.0000	100–120	0.5700	0.4902	0.0200	0.3701	0.9596
20	RM5626	2.0000	190–210	0.8118	0.3056	0.0471	0.2589	0.8477
21	RM3392	4.0000	160–170	0.3416	0.6940	0.4653	0.6320	0.3339
22	RM1278	2.0000	130–140	0.7150	0.4076	0.1500	0.3245	0.6350
23	RM471	2.0000	110–130	0.7500	0.3750	0.1327	0.3047	0.6492
24	RM521	2.0000	240–270	0.9278	0.1340	0.0111	0.1250	0.9180
25	RM6209	2.0000	80–90	0.9798	0.0396	0.0000	0.0388	1.0000
26	RM80	2.0000	135–160	0.6198	0.4713	0.2604	0.3602	0.4516
27	OsZIP8	2.0000	0–100	0.6863	0.4306	0.0000	0.3379	1.0000
28	RM152	3.0000	140–180	0.4023	0.6585	0.5632	0.5845	0.1503
29	RM440	3.0000	150–205	0.7961	0.3438	0.0132	0.3150	0.9622
30	RM432	3.0000	170–350	0.7990	0.3399	0.0882	0.3121	0.7426
31	RM434	3.0000	150–290	0.7677	0.3706	0.0808	0.3233	0.7839
32	RM 3	3.0000	115–160	0.5337	0.5716	0.0337	0.4871	0.9417
33	RM 1	3.0000	75–120	0.7464	0.4101	0.0435	0.3730	0.8954
34	RM 144	3.0000	235–270	0.5000	0.5955	0.1000	0.5137	0.8343
35	RM 201	2.0000	140–160	0.8693	0.2272	0.0568	0.2014	0.7524
36	RM 205	4.0000	100–140	0.6325	0.5547	0.0843	0.5172	0.8497
37	RM 270	2.0000	300–370	0.9412	0.1107	0.0000	0.1046	1.0000
38	RM 335	2.0000	100–110	0.6616	0.4478	0.0303	0.3475	0.9330
39	RM154	3.0000	130–200	0.4765	0.6358	0.4471	0.5641	0.3022
40	RM211	3.0000	120–170	0.6534	0.4884	0.1591	0.4156	0.6774
41	RM202	3.0000	150–200	0.5150	0.6106	0.0900	0.5385	0.8540
42	RM293	2.0000	200–210	0.7557	0.3693	0.0114	0.3011	0.9696
43	RM85	3.0000	90–110	0.5058	0.6182	0.0814	0.5468	0.8698
44	RM407	6.0000	170–550	0.4892	0.7019	0.1290	0.6713	0.8180
45	RM237	2.0000	130–150	0.5345	0.4976	0.0115	0.3738	0.9772
46	RM259	4.0000	180–280	0.6867	0.4806	0.0400	0.4346	0.9178
47	RM421	2.0000	240–300	0.9521	0.0912	0.0532	0.0870	0.4209
48	RM235	3.0000	90–150	0.6462	0.4857	0.2462	0.4044	0.4990
49	RM1337	2.0000	120–140	0.6875	0.4297	0.5000	0.3374	######
50	RM3409	2.0000	304–340	0.7692	0.3550	0.0000	0.2920	1.0000
51	RM105	2.0000	130–145	0.6076	0.4768	0.0000	0.3632	1.0000
52	RM309	1.0000	190–190	1.0000	0.0000	0.0000	0.0000	NaN
53	RM452	1.0000	250–250	1.0000	0.0000	0.0000	0.0000	NaN
54	RM204	3.0000	120–170	0.4695	0.5741	0.0244	0.4807	0.9580
55	RM137	1.0000	250–250	1.0000	0.0000	0.0000	0.0000	NaN
56	RM1789	1.0000	180–180	1.0000	0.0000	0.0000	0.0000	NaN
57	RM6641	2.0000	140–150	0.7527	0.3723	0.0000	0.3030	1.0000
58	RM296	4.0000	140–410	0.5337	0.6233	0.0337	0.5673	0.9465
59	RM3331	2.0000	165–175	0.5306	0.4981	0.0000	0.3741	1.0000
60	RM31	3.0000	100–125	0.5000	0.6157	0.0455	0.5419	0.9270
61	RM429	2.0000	120–140	0.9511	0.0930	0.0326	0.0887	0.6527
62	RM556	2.0000	130–190	0.7157	0.4070	0.0196	0.3242	0.9523
63	RM585	4.0000	130–210	0.3824	0.6915	0.0196	0.6330	0.9719
64	RM23	2.0000	150–160	0.8713	0.2243	0.0000	0.1991	1.0000
65	RM34	2.0000	160–180	0.9188	0.1493	0.0875	0.1382	0.4191
66	RM53	2.0000	180–200	0.5714	0.4898	0.0000	0.3698	1.0000
67	RM300	3.0000	130–150	0.6344	0.5305	0.0000	0.4743	1.0000
68	RM315	2.0000	145–150	0.8687	0.2281	0.0000	0.2021	1.0000
69	RM339	3.0000	150–190	0.7475	0.4081	0.0707	0.3706	0.8283
70	RM400	4.0000	250–340	0.6571	0.5224	0.0000	0.4819	1.0000
71	RM528	2.0000	300–320	0.5464	0.4957	0.0000	0.3728	1.0000
72	RM486	2.0000	135–140	0.7526	0.3724	0.0206	0.3031	0.9452
73	RM340	6.0000	130–290	0.5215	0.6656	0.1828	0.6293	0.7279
74	RM1132	4.0000	95–140	0.5505	0.6145	0.0303	0.5617	0.9512
75	RM441	1.0000	170–170	1.0000	0.0000	0.0000	0.0000	NaN
76	RM590	1.0000	190–190	1.0000	0.0000	0.0000	0.0000	0.0000
77	RM258	1.0000	250–250	1.0000	0.0000	0.0000	0.0000	0.0000
78	GRMM9–1	2.0000	0–275	0.6961	0.4231	0.0000	0.3336	1.0000
79	GRMM9–2	2.0000	0–150	0.9216	0.1446	0.0000	0.1341	1.0000
80	OsNAC	2.0000	0–600	0.7917	0.3299	0.0000	0.2755	1.0000
81	OsZIP8A	2.0000	0–900	0.5882	0.4844	0.0000	0.3671	1.0000
82	OsZIP8C	2.0000	0–930	0.5980	0.4808	0.0000	0.3652	1.0000
83	OsYSL4E	3.0000	0–851	0.5000	0.6250	0.0000	0.5546	1.0000
84	OsMTP1A	2.0000	0–950	0.7451	0.3799	0.0000	0.3077	1.0000
85	OsNRAMP5G	2.0000	0–150	0.6373	0.4623	0.0000	0.3555	1.0000
86	IRMM9–1	2.0000	0–200	0.7059	0.4152	0.0000	0.3290	1.0000
87	OsYSL1	2.0000	0–230	0.6667	0.4444	0.0000	0.3457	1.0000
88	OsYSL2A	2.0000	0–150	0.9216	0.1446	0.0000	0.1341	1.0000
89	OsYSL2B	3.0000	0–330	0.4412	0.6353	0.0000	0.5590	1.0000
90	OsYSL5	2.0000	0–380	0.5098	0.4998	0.0000	0.3749	1.0000
91	OsYSL6	2.0000	0–166	0.5098	0.4998	0.0000	0.3749	1.0000
92	OsYSL11	2.0000	0–190	0.6569	0.4508	0.0000	0.3492	1.0000
93	OsZIP6A	3.0000	0–170	0.5098	0.5352	0.0000	0.4280	1.0000
94	OsZIP6B	2.0000	0–220	0.6078	0.4767	0.0000	0.3631	1.0000
95	OsZIP7	3.0000	0–180	0.6569	0.4796	0.0000	0.4025	1.0000
96	OsZIP8	2.0000	0–160	0.5294	0.4983	0.0000	0.3741	1.0000
97	OsNRAMP1A	3.0000	0–310	0.6667	0.4931	0.0000	0.4364	1.0000
98	OsNRAMP1B	2.0000	0–250	0.6471	0.4567	0.0000	0.3524	1.0000
99	OsFER1	3.0000	0–170	0.8431	0.2737	0.0000	0.2518	1.0000
100	OsFER2	3.0000	0–475	0.4804	0.5992	0.0000	0.5157	1.0000
	Mean	2.44		0.682038	0.414195	0.061385	0.348211	0.849347

### Population structure

**Fig. 3:**
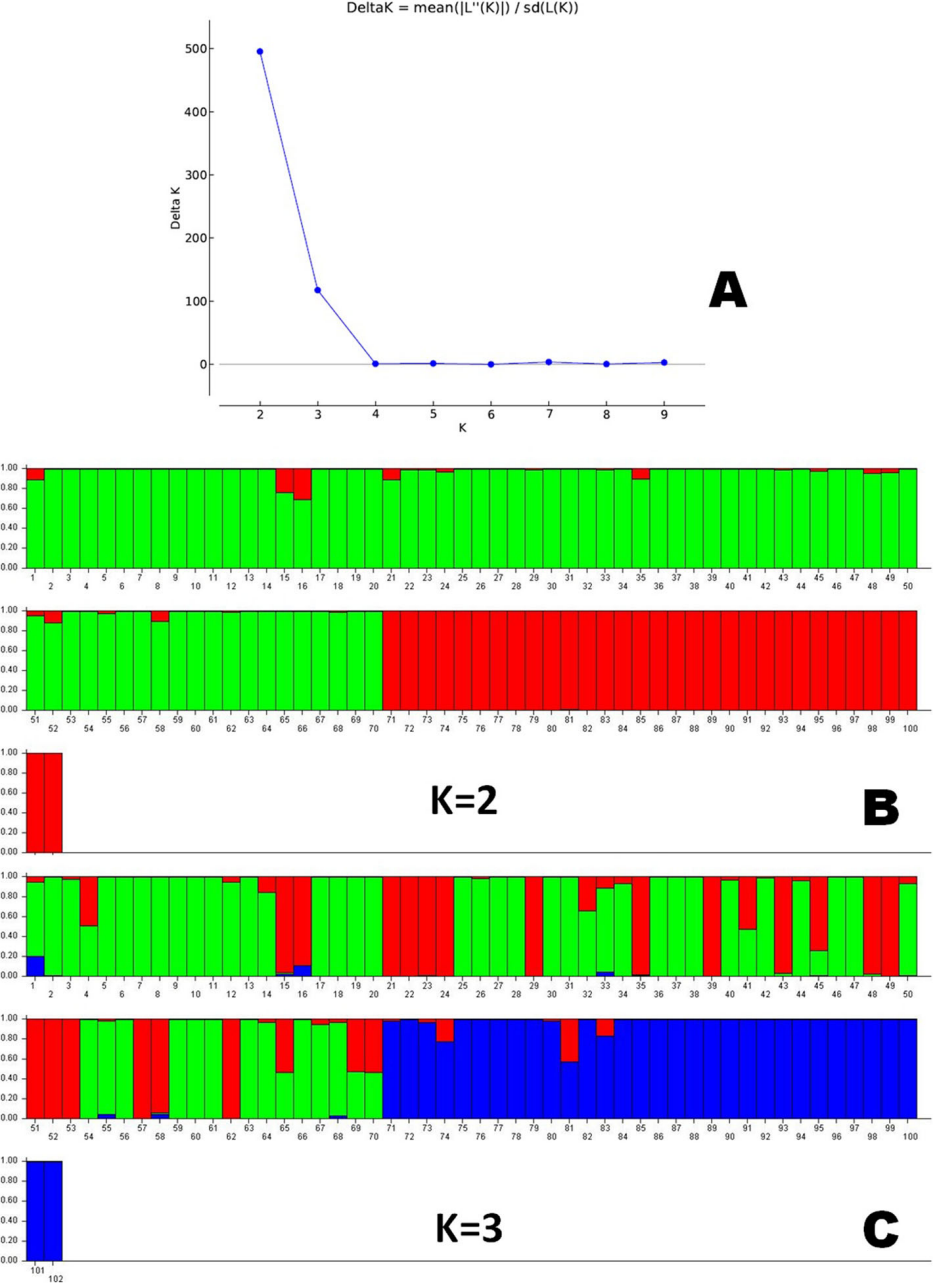
**a** Graph of delta K value, an ad-hoc statistic related to the rate of change in the log probability of data between successive K values; **b** Population structure of the 102-panel population placed based on membership probability fractions of individual genotypes at K = 2 and **c** Population structure of the 102-panel population placed based on membership probability fractions of individual genotypes at K = 3. The genotypes with the probability of ≥80% membership fractions were assigned to corresponding subgroups with others categorized as admixture. The numbers in the figure represent the serial number of the genotypes enlisted in Table 1

A Bayesian clustering approach was followed for estimation of genetic structure by taking probable sub-populations (K) and higher delta K-value using the STRUCTURE 2.3.6 software. The genotypes in the panel exhibiting variation for grain Fe and Zn content were evaluated for genetic structure following. It categorized the genotypes into two sub-populations (Fig. 3a, b) with a high ∆K peak value of 495.2 at K = 2 among the assumed K (Fig. 3a). But, the classification was not robust with respect to Fe-Zn content in the genotypes. However, at K = 3 with ∆K value of 117.9 categorized the population into three distinct sub-populations with perfect grouping. At K = 3, the sub-population 1 (SP1), sub-population 2 (SP2) and sub-population 3 (SP3) included 18, 44 and 40 genotypes, respectively whereas eleven genotypes were admixture type having major genetic constituent from three sub-groups (Fig. 3c; Table 3). The SP1 and SP2 included majority of moderate and high grain Fe-Zn containing genotypes accommodating 100% of total high Fe (> 4 mg kg^− 1^) genotypes, 97.3% of total moderate Fe (3–4 mg kg^− 1^) genotypes, 90% of total high Zn (> 20 mg kg^− 1^) genotypes and 69.2% of total moderate Zn (15–20 mg kg^− 1^) genotypes. Maximum allele frequency divergence between the two sub-populations (net nucleotide distance) 1–2, 1–3 and 2–3 were 0.1239, 0.1813 and 0.2480, respectively. The average distance (expected heterozygosity) between individuals in cluster 1, cluster 2 and cluster 3 were 0.3140, 0.3051 and 0.3115, respectively. The three sub-populations showed fixation index values (F_ST_) of 0.287 for SP1, 0.385 for SP2 and 0.4853 for SP3. A lower value of alpha (alpha = 0.0388) was found for the studied panel population. The distribution pattern of alpha-value in the panel population showed a leptokurtic symmetry while distribution of F_ST_ values in the sub-populations were almost in symmetric shape with same to the left and right from the centre (Additional file 2: Figure S2). Further at K = 7 with ∆K value of 3.946, the software categorized the entire population into 7 distinct sub-populations classifying the population into more subtle classes according to grain Fe-Zn content.

**Table 3:** The inferred ancestry value and population structure in the panel population containing 102 genotypes with their Fe-Zn classification category

Sl. No.	Genotype name/National testing No.	Inferred ancestry at K = 2		Structure group	Inferred ancestry at K = 3			group	Classification of genotype based on Fe & Zn content
		Q1	Q2		Q1	Q2	Q3		
1	IET23829	0.111	0.889	SP2	0.053	0.745	0.202	SP2	H-Fe & M-Zn
2	IR64	0.004	0.996	SP2	0.006	0.985	0.009	SP2	H-Fe & M-Zn
3	IET23834	0.001	0.999	SP2	0.024	0.975	0.001	SP2	M-Fe & M-Zn
4	Kalanamak	0.001	0.999	SP2	0.489	0.51	0.001	SP2	H-Fe & H-Zn
5	IET23824	0.001	0.999	SP2	0.006	0.993	0.001	SP2	M-Fe & H-Zn
6	IET23832	0.002	0.998	SP2	0.001	0.998	0.001	SP2	H-Fe & M-Zn
7	Chittimuthyalu	0.001	0.999	SP2	0.002	0.998	0.001	SP2	H-Fe & H-Zn
8	IET24780	0.001	0.999	SP2	0.001	0.998	0.001	SP2	H-Fe & H-Zn
9	BPT5204	0.001	0.999	SP2	0.007	0.992	0.001	SP2	H-Fe & M-Zn
10	IET24771	0.001	0.999	SP2	0.002	0.997	0.001	SP2	M-Fe & M-Zn
11	IET24766	0.001	0.999	SP2	0.005	0.994	0.001	SP2	M-Fe & M-Zn
12	IET24316	0.001	0.999	SP2	0.057	0.938	0.004	SP2	H-Fe & M-Zn
13	IET24391	0.001	0.999	SP2	0.002	0.997	0.001	SP2	L-Fe & H-Zn
14	IET24777	0.002	0.998	SP2	0.154	0.843	0.003	SP2	L-Fe & L-Zn
15	IET24760	0.242	0.758	SP2	0.96	0.018	0.022	SP1	H-Fe & M-Zn
16	IET24775	0.311	0.689	SP2	0.886	0.005	0.108	SP1	H-Fe & M-Zn
17	IET24783	0.002	0.998	SP2	0.002	0.997	0.001	SP2	M-Fe & M-Zn
18	IET24544	0.001	0.999	SP2	0.003	0.996	0.002	SP2	L-Fe & M-Zn
19	IET24336	0.001	0.999	SP2	0.005	0.994	0.001	SP2	L-Fe & M-Zn
20	IET24772	0.001	0.999	SP2	0.003	0.996	0.001	SP2	M-Fe & M-Zn
21	IET24557	0.111	0.889	SP2	0.993	0.002	0.004	SP1	M-Fe & M-Zn
22	IET24779	0.009	0.991	SP2	0.997	0.002	0.001	SP1	H-Fe & M-Zn
23	IET24774	0.008	0.992	SP2	0.99	0.009	0.001	SP1	L-Fe & M-Zn
24	IET24787	0.032	0.968	SP2	0.996	0.002	0.002	SP1	M-Fe & M-Zn
25	IET25441	0.001	0.999	SP2	0.004	0.994	0.001	SP2	H-Fe & M-Zn
26	IET25443	0.001	0.999	SP2	0.015	0.984	0.001	SP2	M-Fe & M-Zn
27	IET25444	0.001	0.999	SP2	0.001	0.998	0.001	SP2	M-Fe & M-Zn
28	IET25445	0.001	0.999	SP2	0.002	0.997	0.001	SP2	M-Fe & L-Zn
29	IET25446	0.009	0.991	SP2	0.995	0.003	0.002	SP1	M-Fe & H-Zn
30	IET25447	0.001	0.999	SP2	0.002	0.997	0.001	SP2	M-Fe & M-Zn
31	IET25449	0.001	0.999	SP2	0.001	0.998	0.001	SP2	M-Fe & M-Zn
32	IET25450	0.001	0.999	SP2	0.341	0.658	0.002	SP2	H-Fe & H-Zn
33	IET25452	0.012	0.988	SP2	0.112	0.843	0.045	SP2	M-Fe & M-Zn
34	IET25453	0.001	0.999	SP2	0.067	0.93	0.003	SP2	M-Fe & M-Zn
35	IET25454	0.106	0.894	SP2	0.983	0.007	0.01	SP1	M-Fe & M-Zn
36	IET25457	0.001	0.999	SP2	0.002	0.997	0.001	SP2	L-Fe & M-Zn
37	IET25459	0.001	0.999	SP2	0.002	0.998	0.001	SP2	L-Fe & M-Zn
38	IET25460	0.001	0.999	SP2	0.004	0.995	0.001	SP2	M-Fe & L-Zn
39	IET25461	0.007	0.993	SP2	0.994	0.005	0.001	SP1	M-Fe & H-Zn
40	IET25463	0.002	0.998	SP2	0.032	0.965	0.004	SP2	M-Fe & M-Zn
41	IET25464	0.001	0.999	SP2	0.525	0.474	0.001	SP1	L-Fe & M-Zn
42	IET25465	0.001	0.999	SP2	0.009	0.988	0.002	SP2	H-Fe & H-Zn
43	Gontra Bidhan3	0.009	0.991	SP2	0.97	0.028	0.001	SP1	L-Fe & L-Zn
44	IET25469	0.001	0.999	SP2	0.038	0.959	0.003	SP2	M-Fe & M-Zn
45	IET25470	0.022	0.978	SP2	0.736	0.256	0.008	SP1	L-Fe & M-Zn
46	IET25471	0.001	0.999	SP2	0.004	0.994	0.002	SP2	M-Fe & M-Zn
47	DRRH3	0.001	0.999	SP2	0.004	0.992	0.003	SP2	L-Fe & L-Zn
48	IET25472	0.048	0.952	SP2	0.976	0.017	0.007	SP1	M-Fe & H-Zn
49	IET25473	0.042	0.958	SP2	0.996	0.002	0.002	SP1	M-Fe & M-Zn
50	IET25474	0.002	0.998	SP2	0.065	0.926	0.008	SP2	M-Fe & M-Zn
51	IET25475	0.05	0.95	SP2	0.997	0.002	0.001	SP1	M-Fe & H-Zn
52	IET25477	0.121	0.879	SP2	0.995	0.001	0.003	SP1	M-Fe & H-Zn
53	IET25478	0.006	0.994	SP2	0.998	0.001	0.001	SP1	L-Fe & M-Zn
54	IET25479	0.001	0.999	SP2	0.006	0.993	0.001	SP2	M-Fe & M-Zn
55	Lalmeeta	0.022	0.978	SP2	0.019	0.934	0.047	SP2	L-Fe & L-Zn
56	Abhimanyu	0.001	0.999	SP2	0.003	0.995	0.001	SP2	M-Fe & L-Zn
57	Kalobhutia	0.007	0.993	SP2	0.998	0.001	0.001	SP1	M-Fe & L-Zn
58	Sadakajam	0.103	0.897	SP2	0.94	0.016	0.044	SP1	H-Fe & M-Zn
59	Geetanjali	0.001	0.999	SP2	0.002	0.998	0.001	SP2	M-Fe & L-Zn
60	Kakhru	0.001	0.999	SP2	0.001	0.998	0.001	SP2	L-Fe & L-Zn
61	Boanti	0.001	0.999	SP2	0.001	0.998	0.001	SP2	M-Fe & L-Zn
62	Tulsimukul	0.008	0.992	SP2	0.998	0.002	0.001	SP1	M-Fe & L-Zn
63	Kokilpatri	0.001	0.999	SP2	0.001	0.998	0.001	SP2	L-Fe & L-Zn
64	Basmatikarnal	0.001	0.999	SP2	0.034	0.965	0.001	SP2	M-Fe & L-Zn
65	Kalonunia	0.001	0.999	SP2	0.534	0.465	0.001	SP1	M-Fe & L-Zn
66	SafedLuchai2	0.001	0.999	SP2	0.001	0.998	0.001	SP2	M-Fe & M-Zn
67	Bankra	0.001	0.999	SP2	0.051	0.946	0.003	SP2	L-Fe &L-Zn
68	Moongi	0.009	0.991	SP2	0.031	0.935	0.033	SP2	L-Fe & L-Zn
69	Swarnakranti	0.001	0.999	SP2	0.524	0.475	0.001	SP1	M-Fe & L-Zn
70	Kalojeera	0.001	0.999	SP2	0.53	0.47	0.001	SP1	M-Fe & L-Zn
71	Ketekijoha	0.999	0.001	SP1	0.015	0.001	0.984	SP3	L- Fe & M- Zn
72	Maudamani	0.999	0.001	SP1	0.002	0.001	0.997	SP3	L- Fe & L- Zn
73	Tarori Basmati	0.999	0.001	SP1	0.032	0.001	0.967	SP3	L- Fe & M- Zn
74	Mamihunger	0.998	0.002	SP1	0.226	0.001	0.773	SP3	L- Fe & H- Zn
75	Sneha	0.999	0.001	SP1	0.002	0.001	0.998	SP3	L-Fe & H-Zn
76	Savitri	0.999	0.001	SP1	0.002	0.001	0.997	SP3	L Fe & L-Zn
77	CR Dhan 101	0.999	0.001	SP1	0.003	0.001	0.996	SP3	L-Fe & L-Zn
78	CR Dhan 907	0.999	0.001	SP1	0.001	0.001	0.998	SP3	M- Fe & M-Zn
79	CR Dhan 801	0.999	0.001	SP1	0.001	0.001	0.998	SP3	L- Fe & L-Zn
80	Chinikamini	0.999	0.001	SP1	0.015	0.001	0.984	SP3	L- Fe & M-Zn
81	Nuakalajeera	0.989	0.011	SP1	0.422	0.001	0.577	SP3	L- Fe & M-Zn
82	Moti	0.999	0.001	SP1	0.002	0.001	0.997	SP3	L- Fe & M-Zn
83	Nuadhusura	0.999	0.001	SP1	0.169	0.001	0.83	SP3	M- Fe & M-Zn
84	Heera	0.999	0.001	SP1	0.002	0.001	0.997	SP3	L- Fe & M-Zn
85	Jalmagna	0.999	0.001	SP1	0.002	0.001	0.997	SP3	L- Fe & M-Zn
86	AC44756	0.999	0.001	SP1	0.001	0.001	0.998	SP3	L- Fe & M-Zn
87	AC44755	0.999	0.001	SP1	0.001	0.001	0.998	SP3	L- Fe & M-Zn
88	AC44754	0.999	0.001	SP1	0.001	0.001	0.998	SP3	L- Fe & M-Zn
89	AC44753	0.999	0.001	SP1	0.003	0.001	0.996	SP3	L- Fe & M- Zn
90	Swarna-Sub 1	0.999	0.001	SP1	0.004	0.001	0.995	SP3	L-Fe & M- Zn
91	Ranjit	0.999	0.001	SP1	0.002	0.001	0.997	SP3	L- Fe & L- Zn
92	Swarna	0.999	0.001	SP1	0.001	0.001	0.998	SP3	L- F & L- Zn
93	Jaya	0.999	0.001	SP1	0.002	0.001	0.998	SP3	L-Fe & M- Zn
94	Samalei	0.999	0.001	SP1	0.001	0.001	0.998	SP3	L- Fe & L- Zn
95	AC44752	0.999	0.001	SP1	0.001	0.001	0.998	SP3	L- Fe & M- Zn
96	Lalat	0.999	0.001	SP1	0.001	0.001	0.998	SP3	L- Fe & H- Zn
97	MTU1010	0.999	0.001	SP1	0.004	0.001	0.995	SP3	L- Fe & M- Zn
98	Naveen	0.999	0.001	SP1	0.002	0.001	0.997	SP3	L- Fe & L- Zn
99	Satabdi	0.999	0.001	SP1	0.001	0.001	0.999	SP3	L- Fe & M- Zn
100	Pooja	0.999	0.001	SP1	0.001	0.001	0.998	SP3	L- Fe & L- Zn
101	Sarala	0.999	0.001	SP1	0.002	0.001	0.997	SP3	L- Fe & L- Zn
102	Agnisar	0.999	0.001	SP1	0.001	0.001	0.998	SP3	L- Fe & L- Zn

*L-Fe* Low grain Fe content, *M-Fe* Medium grain Fe content, *H-Fe* High grain Fe content, *L-Zn: L-Zn* Low grain Zn content, *M-Zn* Medium grain Zn content, *H-Zn* High grain Zn content

### Analysis of molecular variance (AMOVA) and LD decay plot

The analysis of molecular variance (AMOVA) showed genetic variations between and within the sub-populations at K = 2, K = 3 and K = 7 (Table 4). The genetic variations between and within the two sub-populations (K = 2) was 41% among the populations, 50% among individuals and 9% variation within individuals in the panel population. At 3 sub-populations, 38% of the variation among populations, 52% among individuals and 10% variation within individuals in the panel population. But, the analysis at K = 7 revealed 39% of the variation among populations, 51% among individuals and 10% variation within individuals resulted from the analysis. Wright’s F statistic was used to calculate the deviation from Hardy-Weinberg’s prediction. The F_IS_ and F_IT_ value for all the 100 loci were 0.844 and 0.904, whereas F_ST_ was 0.385 among populations at three sub-populations analysis. The values of F_ST_, F_IS_ and F_IT_ for 7 sub-population level were 0.386, 0.831 and 0.897, respectively. The F_ST_ values at both K = 3 and K = 7 could discriminate the sub-populations from each other revealing differences among themselves. A moderate linearized F_ST_ value of 0.385 at K = 3 and 0.386 at K = 7 were computed for the sub-populations. The F_ST_ values of each sub-population and their distribution pattern showed a clear differentiation among the sub-populations from each other (Additional file 2: Figure S2).

**Table 4:** Analysis of molecular variance (AMOVA) of the sub-populations of panel population at K = 2 and K = 3 for Fe and Zn content in milled rice of 102 genotypes

Source of variation	AMOVA for the three sub-populations at K = 2				AMOVA for the seven sub-populations at K = 3			
	df.	Mean sum of squares	Variance components	Percentage variation	df.	Mean sum of squares	Variance components	Percentage variation
Among populations	1	1192.43	13.175	41	2	743	10.99	38
Among individuals (accessions) within population	100	35.06	16.163	50	99	32.45	14.86	52
Within individuals (accessions)	102	2.74	2.74	9	102	2.74	2.74	10
Total	203			100	203		28.59	100
F-Statistics	Value	*P*-value			Value	*P*-value		
F_ST_	0.411	0.001			0.3858	0.001		
F_IS_	0.855	0.001			0.844	0.001		
F_IT_	0.915	0.001			0.904	0.001		
F_ST_ max.	0.614				0.640			
F’_ST_	0.669				0.601			

**Fig. 4:**
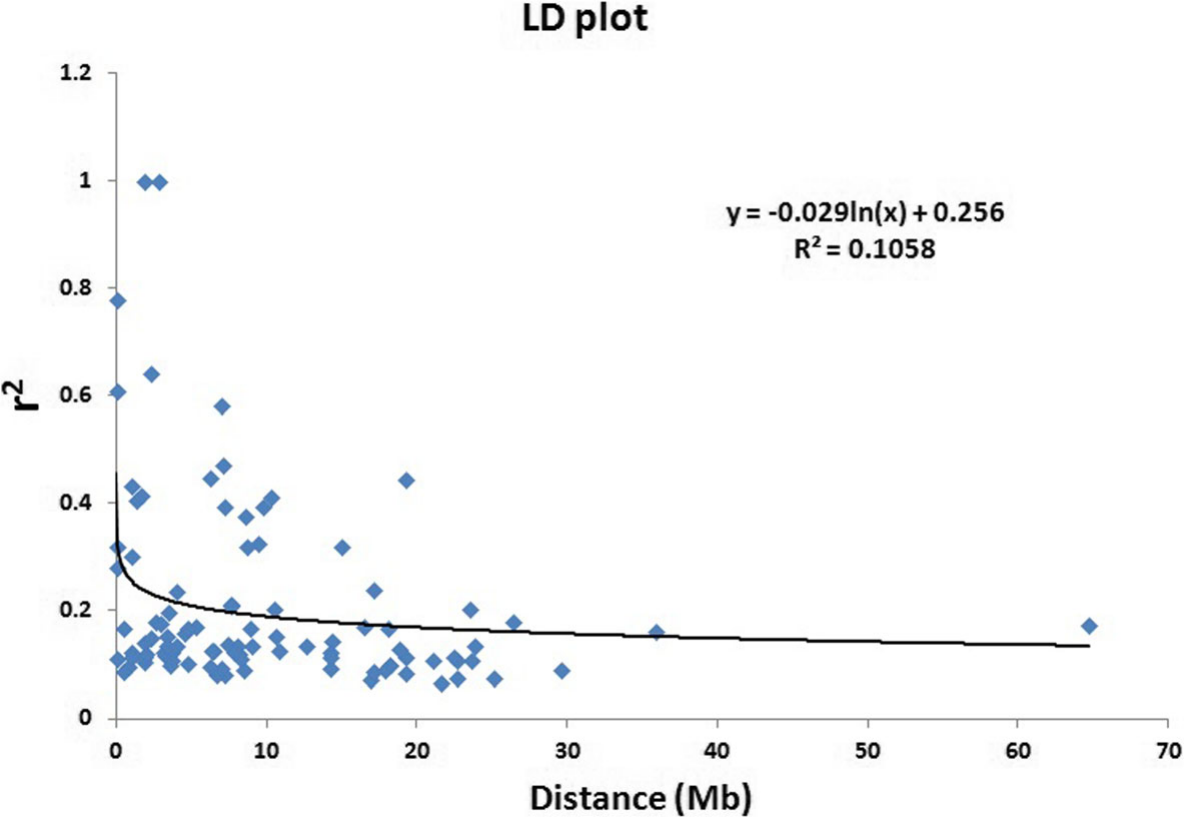
Linkage disequilibrium (LD) decay (*r*^2^) curve plotted against the physical distance (base pairs, bp) between pairs of loci on chromosomes in rice. The decay started in million bp estimated by taking 95th percentile of the distribution of *r*^2^ for all unlinked loci

The linkage disequilibrium decay rate is crucial for maintenance of disequilibrium in a population. This provides scope for mapping of complex traits through marker–trait association. Linkage disequilibrium plot was generated for a population by plotting Syntenic *r*2 values against the physical distance in million base pair. A sharp decline in LD decay was obtained for the linked markers at 1–2 M base pair and subsequently showed a very slow and gradual decay (Fig. 4).

### Grouping of panel population by principal coordinates and cluster analyses

**Fig. 5:**
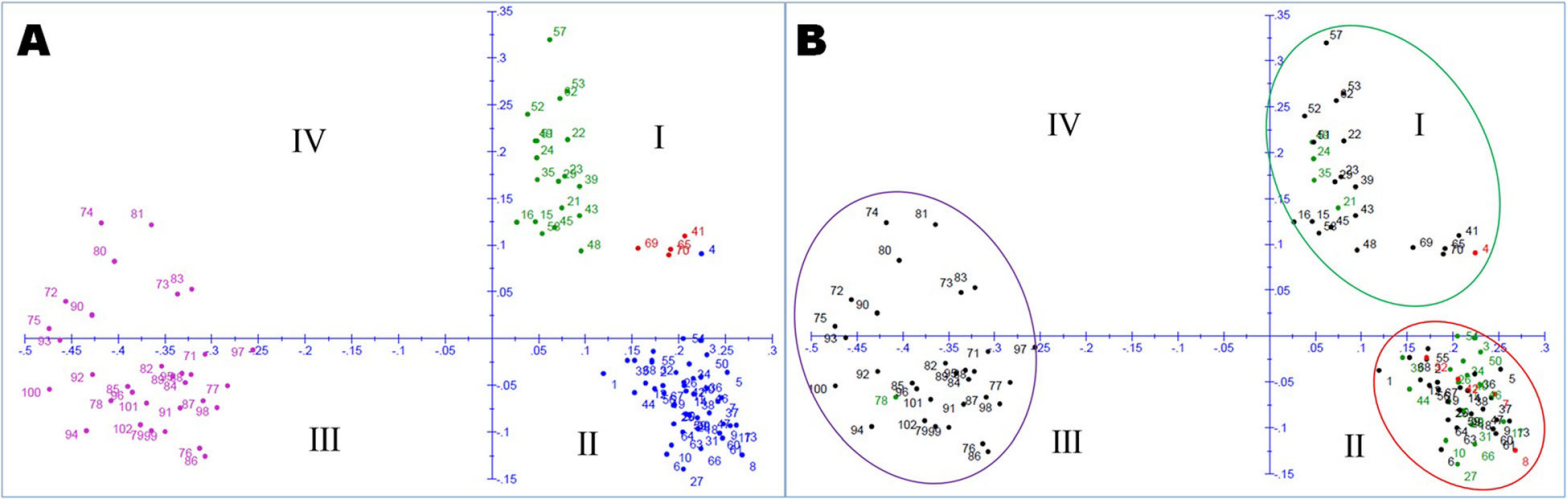
Principal coordinate analysis (PCoA) of 102 genotypes in the panel population for grain Fe and Zn content using 100 molecular markers. The dot numbers in the figure represent the serial number of the genotypes enlisted in Table 1. The numbers are coloured on the basis of (**a**) sub-populations obtained from structure analysis (SP1-green; SP2-blue; SP3-Pink; admix type-red) (**b**) grain Fe and Zn content (common high grain Fe and Zn content – red; common moderate grain Fe and Zn content – green)

Principal coordinate analysis (PCoA) is depicted in two dimension using 100 markers for determining the genetic relatedness among the genotypes (Fig. 5). The first two components accounted for 36.45 and 6.16% of total inertia. The panel population containing 102 genotypes used in the study were distributed in four quadrants showing three major groups (Fig. 5). The 1st, 2nd, 3rd and 4th quadrant consisted of 24, 46, 24 and 8 number of genotypes, respectively. The genotypes belonging to three different sub-populations are grouped in different quadrants. The 2nd quadrant genotypes are divided into two groups of which one group is closer to axis 1 and another is to axis 2. These 2nd group genotypes closer to axis 2 are admix type depicted in red colour (Fig. 5a).

The majority of the genotypes containing moderate to high Fe and Zn content were placed in the 1st (top right) and 2nd (bottom right) quadrants of the PCoA. All the genotypes that are moderate to high for both zinc and iron namely, IET23834, Chittimuthyalu, IET24780, IET24771, IET24766, IET24783, IET24772, IET25443, IET25444, IET25447, IET25449, IET25450, IET25452, IET25453, IET25463, IET25465, IET25469, IET25471, IET25474, IET25479 and Safed Luchai-2 are placed in the red encircled area except five genotypes Kalanamak, IET24557, IET24787, IET25454 and IET25473 located in 1st quadrant (top right) and CR Dhan 907 in the 3rd quadrant (Fig. 5b). The genotypes namely Chittimuthyalu, IET25450, IET25450 and IET25465 containing high zinc and iron are placed in the red encircled area including Kalojeera, Kalonunia, Swarnakranti and IET25464. The green encircled area consisted of mostly the moderate grain Fe and/or Zn containing genotypes, whereas the blue encircled area covering the 3rd and 4th quadrant included mostly the germplasm lines low to Fe and/or Zn content (Fig. 5b).

**Fig. 6:**
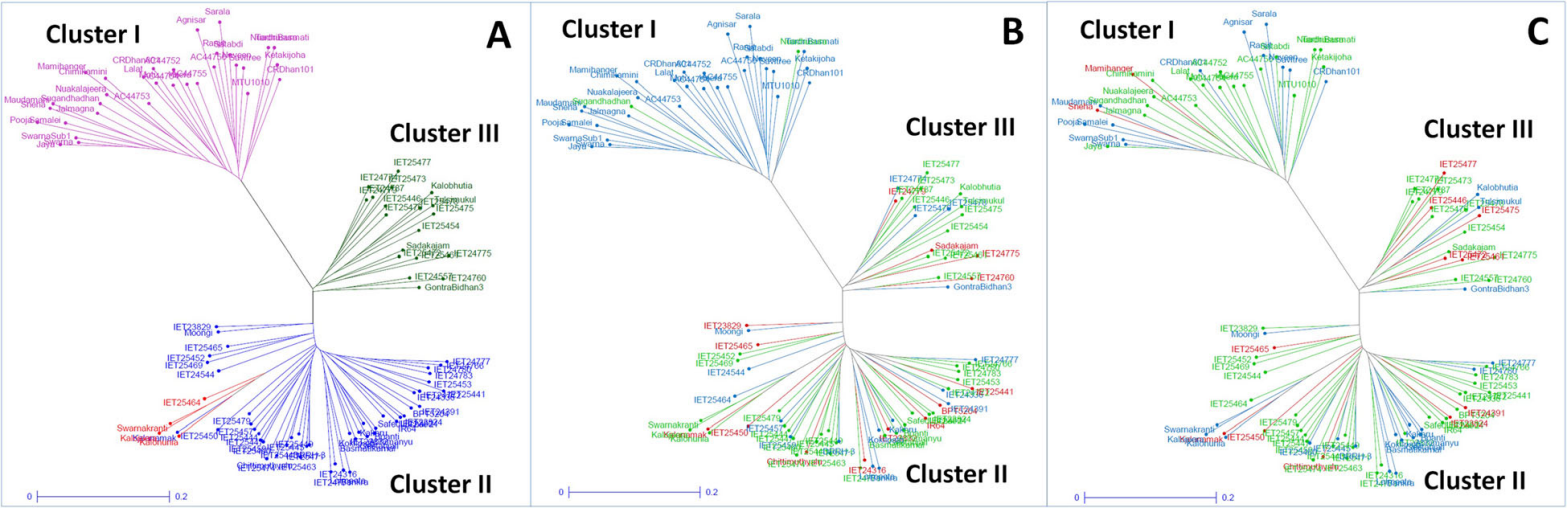
Unrooted tree using unweighted-neighbour joining method depicting clustering pattern of 102 germplasm lines with respect to 100 molecular markers coloured on the basis of (**a**) sub-populations obtained from structure analysis (SP1-green; SP2-blue; SP3-Pink; admix type-red), **b** iron content (High Fe content-red; Moderate Fe content-green; Low Fe content-blue) and **c** Zinc content (High Zn content-red; Moderate Zn content-green; Low Zn content-blue) in milled rice

The cluster analysis based on genotyping of panel population with the 100 markers was performed to determine their collective discrimination ability. The tree constructed with unweighted-neighbour joining method could differentiate the genotypes into 3 different clusters (Fig. 6). The cluster I, II and III consisted of 32, 51 and 19 genotypes, respectively. The three clusters clearly separated the three sub-populations obtained by STRUCTURE analysis. The sub-populations SP1, SP2 and SP3 were grouped separately under cluster III (green), II (blue) and I (pink), respectively (Fig. 6a). The admix genotypes, Kalonunia, Kalanamak, Kalojeera, Swarnakranti, IET25464 formed a distinct sub-cluster under cluster II remaining together with SP2. Significant correlation was observed between the iron and zinc content of rice grain and clustering pattern (Fig. 6b, c). The cluster depicted in red color indicate high, green for moderate and blue for low iron/zinc content in the tree. When iron content was compared, cluster I consisted 30 low iron and 2 (CR Dhan 907 and Nuadhusura) moderate in iron containing genotypes, Cluster III included majority of the high and moderate Fe containing genotypes. Of the 51 genotypes in the cluster II, more than 80% are low to moderate for grain Fe content. Hence, cluster II and III can be classified as moderate to high Fe containing group. Similarly, when Zn content of rice grain was compared with clustering pattern, cluster II and III included majority of the genotypes with high Zn (Fig. 6c). In cluster III, only three genotypes out of 19 genotypes were low for Zn content, where rest were moderate to high Zn. Six genotypes with high and 28 genotypes with moderate Zn content were included in the cluster II of the tree (Fig. 6c).

### Association of marker alleles with grain Fe and Zn content in rice

**Fig. 7:**
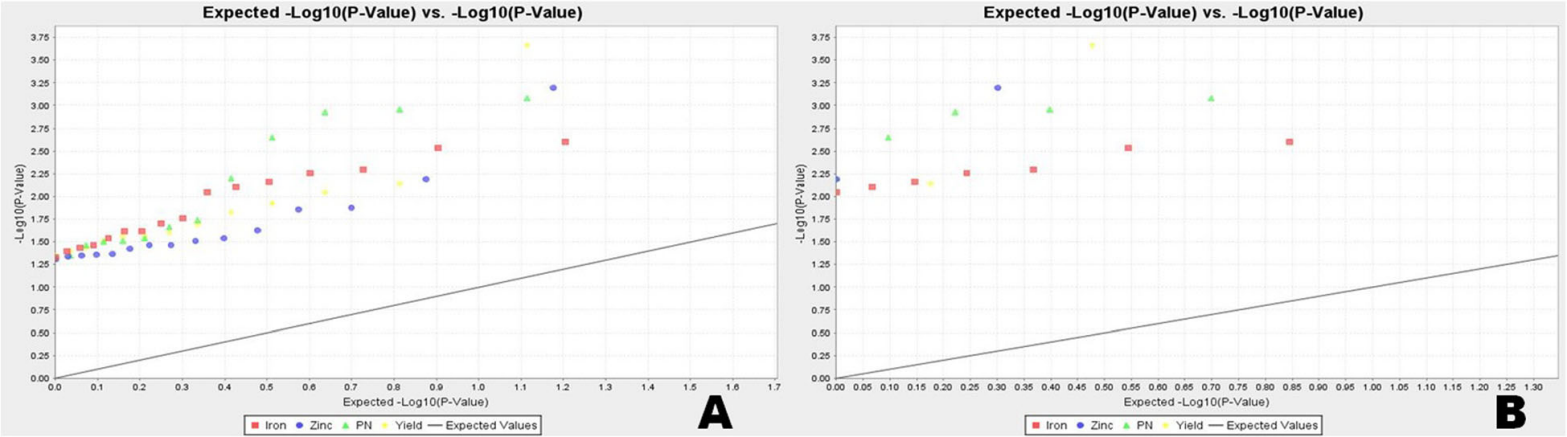
Quantile–Quantile (Q-Q) plot and distribution of marker-trait association from Generalized Linear Model analysis for grain iron-zinc content, panicles/m^2^ and grain yield at (**a**) *p* < 0.01 and (**b**) at *p* < 0.05

Association of grain Fe, Zn content, panicles/m^2^ and grain yield with the molecular markers was estimated using TASSEL 5 software. The association parameters were based on Generalized Linear Model (GLM) and Mixed Linear Model (MLM/ K + Q model). The marker-trait comparisons were subjected to filtration at less than 5% error i.e. 95% confidence (*p* value < 0.05). The GLM approach computed average r^2^ value of 0.1133 with upper limit of 0.6095 and lower limit of 0.0386, whereas MLM showed r^2^ values ranging from 0.0392 to 0.146 with an average of 0.0622 (Additional file 5: Table S3; Additional file 6: Table S4). With r^2^ > 0.10 and *p* ≤ 0.05 filter, 42 and 3 markers showed association with grain Fe and Zn content respectively by using GLM model (Additional file 5: Table S3). However, taking MLM model, only one marker GRMm9–1 showed association with grain Zn content (Table 5). But when r^2^ > 0.05 was taken into account, 11 (RM243, RM122, RM234, RM80, RM315, RM339, RM1132, GRMM9–1, OsZIP8A, OsYSL2A, OsZIP6A) and 3 (RM80, RM300, RM1132) markers were associated with grain Fe and Zn content, respectively (Additional file 5: Table S3; Additional file 6: Table S4). Considering both the GLM and MLM, ten and seven markers showed significant association with grain Fe and Zn content, respectively at *p* < 0.05 (Table 5). Four primers namely RM80, RM300, RM1132 and GRMM9–1 and six primers viz. RM243, RM234, RM80, RM339, OsZIP8B, OsZIP6A were associated with grain Zn and Fe content, respectively with r^2^ > 0.05 and p ≤ 0.05 filter in both the models (Table 5). The Q-Q plot also confirmed the association of the markers with grain Fe and Zn content in rice (Fig. 7).

**Table 5:** Association of marker alleles with Fe and Zn content in milled rice detected both in GLM and MLM analyses in a shortlisted panel population of 102 genotypes

Trait	Marker	GLM				MLM			
		*F* value	*P* value	R^2^	*q* value	*F* value	*P* value	R^2^	*q* value
Fe content	RM243	6.21777	0.01429	0.05854	0.01958259	5.85605	0.01733	0.05798	0.03987069
	RM122	4.3147	0.04035	0.04136	0.04265571	7.11419	0.00892	0.07044	0.027565
	RM234	16.81381	8.41E-05	0.14394	0.00062208	8.05498	0.0055	0.07975	0.027565
	RM7	8.87945	0.00362	0.08155	0.00558083	4.05058	0.04684	0.0401	0.04814111
	RM168	14.70112	2.20E-04	0.12817	0.00093926	4.93231	0.02862	0.04883	0.03987069
	RM80	16.39877	1.01E-04	0.14088	0.0006253	7.60915	0.00691	0.07534	0.03987069
	RM339	11.69699	9.08E-04	0.10472	0.00176731	5.24734	0.02408	0.05195	0.03987069
	RM1132	4.14297	0.04445	0.03978	0.04568472	5.61267	0.01975	0.05557	0.03987069
	OSZIP8	11.44893	0.00102	0.10273	0.001887	5.2546	0.02398	0.05203	0.03987069
	OSZIP6A	5.86773	0.01722	0.05543	0.0205529	7.37666	0.00779	0.07304	0.027565
Zn content	RM260	5.99267	0.01611	0.05654	0.0205529	4.6087	0.03423	0.04563	0.04155935
	RM80	11.86398	8.38E-04	0.10606	0.00172177	5.28924	0.02354	0.05237	0.03987069
	RM300	11.98471	7.91E-04	0.10702	0.00172177	7.74249	0.00645	0.07666	0.027565
	RM339	5.32614	0.02307	0.05057	0.02586636	4.1887	0.04332	0.04147	0.04714235
	RM340	5.59171	0.01998	0.05296	0.02310188	4.78072	0.03111	0.04733	0.03987069
	RM1132	9.0313	0.00335	0.08283	0.00538913	6.3645	0.01322	0.06301	0.03762615
	GRMM9–1	13.00392	4.87E-04	0.11507	0.00137370	12.41426	6.44E-04	0.12291	0.008732
Panicles /m^2^	RM248	18.23846	4.45E-05	0.15425	0.00041122	5.4383	0.0217	0.05384	0.03987069
	RM17	14.62367	2.28E-04	0.12758	0.00093926	11.26493	0.00112	0.11153	0.008732
	RM3392	9.9083	0.00217	0.09015	0.00364954	4.57817	0.03482	0.04533	0.04155935
	RM440	13.87657	3.23E-04	0.12186	0.00119617	4.78325	0.03107	0.04736	0.03987069
	RM85	12.94277	5.01E-04	0.1146	0.00137370	3.98538	0.04862	0.03946	0.04862
	RM421	7.28154	0.00818	0.06787	0.0121064	11.14884	0.00118	0.11038	0.008732
	RM31	4.76448	0.03139	0.04548	0.03415971	4.77291	0.03125	0.04726	0.03987069
	RM556	18.72712	3.58E-05	0.15773	0.00041122	4.90142	0.02911	0.04853	0.03987069
	RM23	15.80571	1.33E-04	0.13648	0.00070178	4.12242	0.04497	0.04082	0.04753971
	RM340	12.95157	4.99E-04	0.11466	0.00137370	5.74925	0.01835	0.05692	0.03987069
	OsNRAMP1A	26.69468	1.22E-06	0.2107	3.206975e-05	11.87069	8.35E-04	0.11753	0.008732
	OsFER1	25.83769	1.73E-06	0.20533	3.206975e-05	9.85209	0.00223	0.09755	0.01375167
Yield/plot	RM243	4.05979	0.0466	0.03901	0.0466	4.3019	0.04064	0.04259	0.04556606
	RM248	5.92531	0.0167	0.05594	0.0205529	5.01002	0.02742	0.0496	0.03987069
	RM17	11.91911	8.16E-04	0.1065	0.00172177	7.11104	0.00894	0.07041	0.027565
	RM517	12.86597	5.20E-04	0.11399	0.00137370	14.7466	2.16E-04	0.14601	0.027565
	RM421	6.89217	0.01002	0.06448	0.01425923	5.55647	0.02036	0.05501	0.03987069
	RM34	6.06991	0.01546	0.05723	0.02042929	5.16896	0.02513	0.05118	0.03987069
	RM339	11.30774	0.00109	0.10159	0.00192047	4.47416	0.0369	0.0443	0.04266563
	RM1132	11.952	8.03E-04	0.10676	0.00172177	4.78822	0.03098	0.04741	0.03987069

Agronomy traits like panicles/m^2^ and grain yield also showed significant association with 44 and 25 primers, respectively using GLM at *P* < 0.05 (Additional file 5: Table S3). The numbers reduced to 13 for each trait when analyzed with MLM model (Additional file 6: Table S4). However, considering GLM and MLM, twelve and eight markers showed significant association with panicles/m^2^ and grain yield, respectively at *p* < 0.05. Of these, nine markers (RM248, RM17, RM440, RM85, RM556, RM23, RM340, OsNARMP1A, OsFER1) with GLM and four markers (RM17, RM421, OsNARMP1A, OsFER1) with GLM as well as MLM showed r^2^ > 0.10 for panicles/m^2^. Similarly, for yield trait, RM17, RM517, RM339 and RM1132 were associated with r^2^ > 0.10 using GLM and that with both the models only RM517 showed r^2^ > 0.10 (Table 5). The Q-Q plot also confirmed the association of the markers with grain panicles/m2 and grain yield in rice (Fig. 7).

Common markers were observed to be associated with different traits. Sixteen and four common markers were observed for Fe and Zn content using GLM and MLM model respectively (Additional file 5: Table S3 and Additional file 6: Table S4). This number reduced to three (RM80, RM339, RM1132) when both the models were considered simultaneously. Considering GLM model, 12 markers viz., RM243, RM488, RM248, RM17, RM3392, RM440, RM201, RM421, RM585, RM34, RM339 and RM1132 showed significant association with all the traits viz., grain Fe and Zn content, panicles/m2 and yield. RM1132 and RM339 were commonly associated with grain Fe, Zn and yield considering both GLM and MLM models (Table 5). OsFER1 was associated with all the traits except grain yield.

### Quantitative real time– PCR for validation of associated markers/genes with grain Fe and Zn content

**Fig. 8:**
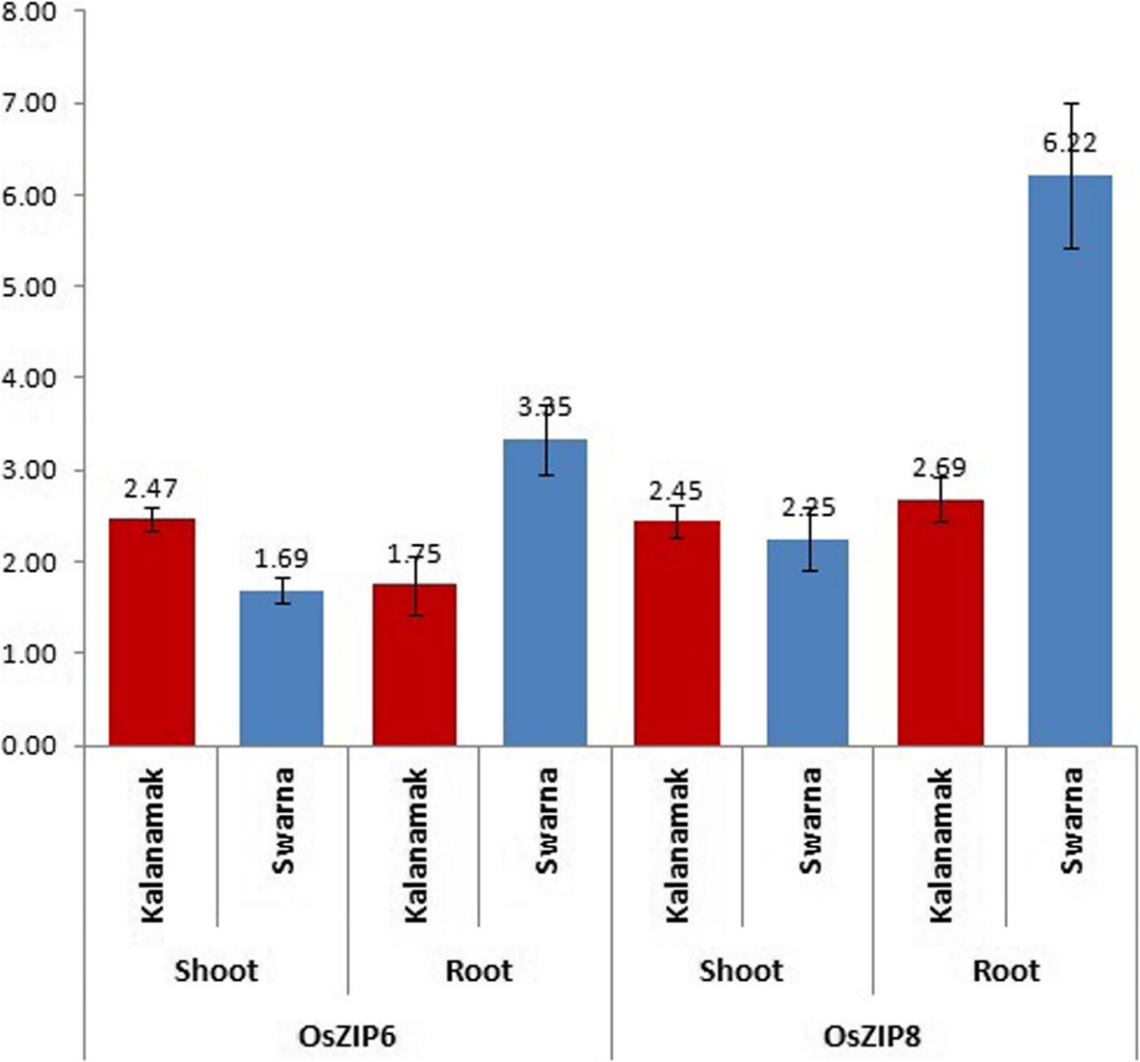
Fold change of expression of genes OsZIP6 and OsZIP8 under Fe-Zn deficient treatment as compared to that of normal condition in roots and shoots of two contrasting genotypes Kalanamak and Swarna

The qRT-PCR analysis was performed for validating the identified markers in the marker-trait association analysis. Two significantly associated markers OsZIP8B and OsZIP6A for the genes OsZIP8 and OsZIP6 respectively were selected for validation study. The results of qRT-PCR for the genes were normalized with housekeeping gene b-tubulin. The expression of the genes was studied in root as well as shoot of two genotypes Kalanmak (high grain Fe and Zn) and Swarna (low grain Fe and Zn) under Fe-Zn normal and deficient condition. Both the genes were up-regulated under Fe-Zn deficient condition as compared to the control situation (Fig. 8). The fold change of expression of both the genes ranged 1.69–6.22 in deficient condition. Higher expression was obtained in root. Swarna showed more upreglation in roots as compared to Kalanamak.

## Discussion

Modern high yielding varieties are poor in essential micronutrients like grain Fe and Zn content in rice [16, 17]. On an average, polished rice has 2 mg kg^− 1^ Fe, while the recommended dietary intake for humans is 10–15 mg kg^− 1^. Previous germplasm study for grain Fe-Zn content reported variability range of 6.3–24.4 mg kg^− 1^ for Fe and 13.5–28.4 mg kg^− 1^ for Zn in rice grain strongly suggests existence of genetic variability of these two micronutrients in rice germplasms [6, 8, 9, 25, 52, 53]. Evaluation of the panel population containing 102 genotypes showed a maximum value of 4.48 mg kg^− 1^ Fe and 26.96 mg kg^− 1^ Zn content in the milled rice (Table 1). Further, biplot analysis revealed a wide variability in Fe and Zn content in the milled rice of the panel population (Fig. 2). It is observed that among the genotypes with > 4 mg kg^− 1^ Fe in milled rice showed > 12 mg kg^− 1^ in brown rice (Table 1). Similarly, the genotypes containing > 20 mg kg^− 1^ Zn in milled rice showed > 25 mg kg^− 1^ in brown rice. Therefore, mapping of QTLs responsible for high Fe and Zn content in the materials seems to be logical. Soil characteristics at the growing locations and genotype x environment has a great role in determining the grain micronutrient content in rice [17, 27]. Therefore, 2 years pooled data for phenotyping of grain Fe and Zn content is more reliable than single year. On the basis of 2 years mean data, the panel population was grouped into three classes based on their grain Fe and Zn content. Therefore, the population structure of the panel population for these traits is very important. Prichard’s structure analysis at K = 3 also grouped the population into three distinct sub-populations.

Further, it was observed that a comparable grain Fe and Zn content were obtained in some varieties and biofortified lines as in the positive check varieties (Table 1). A positive correlation of Fe and Zn content in rice grain in some genotypes had been reported in previous publications [54–56]. Mean performance table generated here also revealed that few genotypes with high Zn content possessed high Fe content also (Table 1). Besides, evaluation results showed that some genotypes were with at par yield with the yield check varieties and contain moderate to high Fe-Zn content in the grains. Therefore, improvement of Fe and Zn content in rice grain along with high grain yield is possible. Relationship of high grain yield with Zn content is reported in earlier publication [57]. A similar observation for grain protein content has been reported recently in rice [17]. From panel’s genotype-trait biplot analysis, it is clear that I^st^ and 2nd quadrant accommodated genotypes with better in Fe and Zn content along with better grain yield (Fig. 2). Previous mapping results also reported linkage of primer RM490 for Zn content associated with yield component QTLs like panicles/plant and 1000-grain weight [58]. Current association mapping revealed association of RM260 with Zn content in rice grain. Also, *qDTY12.1* an-yield QTL under drought was reported to be linked to primer RM260 [36]. The same is associated with Zn content in rice grain (Table 5). Therefore, improvement of Fe-Zn content along with high grain yield is challenging but possible.

The principal component and coordinate analyses distributed the panel’ population according to their grain Fe-Zn content along with grain yield and allocated distinct spots in the four quadrants (Figs. 2 and 5). Distribution of genotypes into different quadrants indicated the presence of genetic variation for Fe and Zn content in the panel population. The neighbour joining tree also grouped the germplasms into different classes on the basis of genotyping results of 100 molecular markers (Fig. 6). The figure showed various groups and sub-groups in the panel population based on genotyping using 100 markers for these traits clearly categorized the panel population into different groups indicating involvement of different genes/QTLs for these classes (Fig. 6). Presence of these different groups in the population increased the continuation of linkage disequilibrium and favoured for getting the marker-trait association for Fe-Zn content in rice grain. Similar results of trait mapping through marker-phenotype association were reported earlier for various rice phenotypic traits [17, 39, 40, 43, 45, 47]. A moderate level of genetic diversity was observed in the studied population for grain Fe and Zn content. The moderate genetic diversity detected in this study for Fe and Zn content is similar to the earlier results on genetic diversity obtained by other workers for these traits [53, 59–61]. However, rich genetic diversity values for these traits were also reported earlier in rice [6, 7, 9, 16, 62–64]. In our structure analysis, we observed a lower value of alpha (α = 0.0388) indicating that these two traits had a common primary ancestor. Subsequently, it evolved with each sub-population with few admix genotypes. The values obtained from inferred ancestry indicated small effects of individual QTLs responsible for trait groups and subgroups in the population. These small effects QTLs need to be put together into a favourable high yielding background through molecular breeding approaches. Similar observations are available from earlier association studies also support this suggestions for gene(s) / QTLs stacking in popular varieties [17, 39, 40, 47].

The F_ST_ values estimated for the pair of sub-populations showed a clear variation in the values. The F_ST_ value for SP1 and SP2 was found to be moderate (0.247) value. The variation in F_ST_ values and their pattern of distribution revealed a clear cut differentiation among the sub-populations from each other with respect to Fe and Zn content in the grains. Thus, the population is categorized into clear genetic groups based on the inter and intra-sub F_ST_ values. It is therefore expected that the populations and individuals showing higher F_ST_ values may produce better progenies containing Fe and Zn content in recombination breeding. Therefore, attempt to pyramid these QTLs responsible for higher Fe and Zn may be put from different populations lead to higher Fe and Zn content in the progenies. Similar suggestions were also provided by earlier workers for increasing high and low temperature stress tolerance and grain yield in rice [17, 39, 40, 65]. A sharp decline and then very slow and gradual LD decay value was noticed in the panel population (Fig. 4). This may be due to the self pollination nature of rice crop. The LD decay is usually high in open pollinated crops.

TASSEL analysis detected marker and grain Fe-Zn content association in the tested panel population. Higher F and lower *p* value with high *r*^*2*^ were detected for 10 and 7 markers associated with Fe and Zn content in kernel, respectively. These were the reliable markers to be used in Fe and Zn enhancement breeding program as detected through both GLM and MLM analyses. Thus, suggesting a strong association of marker with grain Fe-Zn content in the panel population. This is further evident from Q-Q plot exhibiting expression of many QTLs for grain Fe and Zn content (Fig. 7). This marker-trait association provides clue for presence of various QTLs in the germplasms containing more grain Fe and Zn. It may be due to the involvement of different donor lines carrying different QTLs for these two traits. The accumulation of useful QTLs for these traits in few landraces may be possible by natural introgression over a long time period ultimately exhibiting a higher quantity of the two micronutrients in those germplasm lines. Similar predictions for accumulation of useful QTLs in stress tolerant wild and landraces genotypes were also earlier reported in rice [17, 39, 40].

All the significantly associated markers for grain Fe content namely RM243, RM80, RM339, OsZIP6A and OsZIP8B detected through both GLM and MLM models with r^2^ > 0.05 and high F value indicated a very strong association. Probably, these QTLs contribute to variability for Fe content in rice grain. Some of these markers were reported to be linked to Fe enhancing QTLs in rice. Similarly, four markers namely RM80, RM300, RM1132 and gRMm9–1 showed significant association with Zn content detected through both the models at r^2^ > 0.05. These primers are located in different chromosomes which may correspond to different QTLs (Table 5).

Ten markers namely RM243, RM122, RM234, RM7, RM168, RM80, RM339, RM1132, OsZIP6A and OsZIP8B showed significant association with grain iron content detected with both GLM and MLM models. RM243, RM168, RM122, RM234 and RM80 are earlier reported for *qFe1.1*, *qFe3.1*, *qFe5.1*, *qFe7.1* and *qFe8.1* QTLs, respectively [9]. The marker RM339 is physically located closer to RM80 (*qFe8.1*), it will be useful in marker-assisted transfer of *qFe8*.1. Hence, these six markers and five QTLs were validated using the panel population and these reliably can be deployed in MAS breeding for improvement of Fe content in rice grain. OsZIP8 and OsZIP6 are Zinc-iron transporters belonging to ZIP family of micronutrient transporters. Particularly, OsZIP8 is important for Zn transport to seed [66]. Both these transporters being associated with grain Fe and Zn content in our study imply that both of these genes are also responsible for Fe transport to seed. These two genes were also reported earlier and currently detected by both the models [8, 28, 66]. Therefore, these two markers can reliably be used for Fe enhancement. Two markers RM7 and RM1132 were reported earlier for *qZn3.1* and *qZn7*, respectively for grain Zn content [9, 67]. However, these two primers were now strongly associated for grain Fe content detected by both the models. QTLs controlling Fe content located in these locations of the two chromosomes were not reported earlier. Hence, these two locations can be designated as QTLs *qFe3.3* and *qFe7.3* and are novel QTLs. Two primers, RM340 and RM1132 reported for *qZn6* and *qZn7* are now detected to be strongly associated with grain Zn content [67]. Hence, these two QTLs are validated and reliably may be deployed for Zn enhancement in rice grain. Two markers namely RM339 and gRMm9–1 (LOC_Os09g27330: oxidoreductase/ transition metal binding protein) were reported earlier and currently detected by both the models [8, 15]. Therefore, these two markers can also reliably be used for Zn enhancement. Two primers RM80 and RM260 were reported earlier for *qFe8.1* and *qFe12.2* for grain Fe content [9]. However, these two primers under this study were strongly associated for grain Zn content detected by both the models. QTLs controlling Zn content present in these regions of the chromosomes were not reported in previous publications. Hence, these two positions on chromosome 8 and 12 are designated as QTLs *qZn8.3* and *qZn12.3* and considered to be novel QTLs controlling grain Zn content in rice. RM300, located on chromosome 2, is detected to be strongly associated with Zn content. No earlier reports could be found, hence, designated as *qZn2.2* that controls grain Zn content. From mapping study, it is reported that RM1132 is associated with grain Zn content with *qZn7* controlling the trait [67]. However, we detected a strong association for grain Fe content in the same location on the chromosome 7. As these locations on chromosome 7, 8 and 12 are commonly responsible for Zn and Fe content, is can be said that these Fe and Zn enhancing QTLs may be co-localized.

Several markers strongly associated with grain Fe and/or Zn content were observed with single model (GLM or MLM). The different markers used for zinc/iron transporters belonging to ZIP family, yellow stripe gene family (YSL), ferritin, metal tolerance protein (MTP) family, Natural Resistance Associated Macrophage protein 1 (NRAMP) family were associated with Fe content indicating common transport mechanism for Fe and Zn. Ferritins are iron storage proteins having two functional units for rapid uptake of Fe^2+^ and nucleation of Fe^3+^ [68, 69]. Association of OsFER1 with Zn content and panicle number may imply the involvement of this gene with Zn storage along with Fe thereby increasing panicle number of plants as both are essential micronutrients for plant growth. As plant ferritins lack iron response elements [69]. There may be chance of carrying Zn along with Fe. But this needs further confirmation with detailed experimentation.

Markers associated with Fe and Zn were analysed to see their involvement in enhancement of yield and its component traits. Twelve markers viz.*,* RM243, RM488, RM248, RM17, RM3392, RM440, RM201, RM421, RM585, RM34, RM339, RM1132 showed significant association with grain Fe and Zn content as well as panicles/m^2^ and yield considering GLM model only (Additional file 5: Table S3). This indicated that the QTLs controlling these four traits may be co-localized and hence higher chance of co-inheritance. Similarly two markers RM1132 and RM339 were commonly associated with grain Fe, Zn and yield detected considering both GLM and MLM (Table 5). The QTLs controlling grain weight, *qTBGW8* [70] and plant architecture, *OsSPL14* [71] are located only 0.46 Mb and 7.33 Mb apart, respectively, from RM339 located on chromosome 8. Also, RM1132 is located only 5.1 Mb away from *qPN7* reported to control panicle number of rice plant [70]. These observations provide clues for common inheritance of these QTLs. Hence, enhancement of both the traits viz., grain Fe and Zn content and yield parameters can easily be achieved simultaneously. Also, in a recent publication, it is established that grain Fe-Zn can easily be improved with few yield component traits [36]. Similar was the case of grain protein content heritability, negative relationship with grain yield and influence of genotype and environment interaction [18–21]. However, the recent results published on protein content mapping in rice indicated about the possibility of improving grain protein content along with high grain yield [17].

The qRT-PCR analysis was performed for validating the identified markers in the marker-trait association analysis. The expression of the two studied genes OsZIP8 (Fe-Zn transporter) and OsZIP6 (Zn transporter) under Fe-Zn normal condition and their upregulation in deficient condition confirmed the accuracy of the association study. Hence, it can be concluded that other QTLs significantly associated will be useful. Both OsZIP8 and OsZIP6 were up-regulated under Fe-Zn deficient condition as compared to the control situation (Fig. 8). Earlier studies also reported higher expression of these genes under deficient condition [72, 73]. Higher expression of both the genes in roots of Swarna (low grain Fe and Zn) under deficient condition indicates more uptake of the nutrient in the roots but it may not be getting properly channelized to the grain as evident from lower upregulation in shoots of Swarna, It might be utilising the nutrient more for its survival rather storing in the grains. But in case of Kalanamak (high grain Fe and Zn), both the genes showed almost equal up-regulation in roots and shoots thereby translocating Zn and Fe to the grain.

## Conclusion

Polished rice consumed in India is very low in iron and zinc micronutrient content. Phenotyping results categorized genotypes based on grain Fe and Zn content each into 3 sub-classes. Evaluation results indicated that 3 genotypes namely IET24779, IET25465 and Chittimuthyalu produced at par yield with standard check variety IR64 along with high grain Fe-Zn content. Therefore, it is possible to improve grain Fe and Zn content in rice along with high grain yield. However, the studied panel showed a moderate level of genetic diversity for Fe and Zn content based on 100 molecular markers. Linkage disequilibrium for Fe and Zn content was observed in the studied population showing deviation from Hardy-Weinberg’s expectation based on F statistic values. The analysis detected 38% of the variation among populations, 52%among individuals and 10% within individuals in the studied panel population. The panel population was categorized into three sub-populations by the STRUCTURE analysis. The analysis also revealed a common primary ancestor for each sub-population with few admix individuals. Association mapping detected 10 QTLs for grain Fe and 7 for grain Zn content to be strongly associated through both Generalized Linear Model (GLM) and Mixed Linear Model (MLM). Novel QTLs namely *qFe3.3* and *qFe7.3* for grain Fe and *qZn8.3* and *qZn12.3* for grain Zn content were detected. Four QTLs controlling grain Fe and Zn were detected to be co-localized on the same chromosome. Besides, some Fe-Zn controlling QTLs were detected to be co-localised with the yield and its component QTLs. Incorporation of these QTLs may enhance grain yield too. The strongly associated markers with QTLs controlling high grain Fe-Zn namely *qFe1.1*, *qFe3.1*, *qFe5.1, qFe7.1, qFe8.1, OsZIP8B (Zn-Fe regulated transporter), OsZIP6A (LOC_Os05g0164800), qFe3.3 and qFe7.3 for Fe and qZn6, qZn7, qZn2.2, gRMm9–1 (*LOC_Os09g27330*), qZn8.3* and *qZn12.3* will be useful QTLs in stacking for developing nutrient dense rice.

## Methods

### Plant materials and field experiment

A total of 485 germplasm lines were screened for Fe and Zn content of milled rice during 2013 to 2015 [74–76]. The landraces and cultivars were obtained from ICAR- National Rice Research Institute (NRRI), gene bank while the Fe-Zn biofortified lines were from national coordinated trials. A panel population containing 102 shortlisted genotypes representing the three distinct phenotypic classes for grain Fe-Zn content and three check varieties were included in the panel for association study of the micronutrient contents in rice (Additional file 3: Table S1). The cultivars, Kalanamak and Chittimuthyalu for high Fe-Zn content while IR64 for grain yield were used as checks. The genotypes were planted in randomized block design with three replications during wet season, 2016 and 2017 at ICAR-NRRI, Cuttack following a standard procedure for a good crop. Phenotypic performances of the genotypes for days to 50% flowering, panicles/m^2^, iron and zinc content in milled rice and grain yield were recorded during both the years. Data analyses of the traits were performed using the software CROPSTAT v7.0.

### Phenotyping for grain iron and zinc content in rice

Each harvested paddy sample was processed to white rice using Krishi international H-810 non-ferrous Huller and Krishi international K-710 polisher. Before dehusking, the grains were washed with 0.1 N HCl followed by rinsing with double distilled water to make the sample free from contamination. Iron and zinc content (mg/Kg) were determined at ICAR-Indian Institute of Rice Research, Hyderabad using energy dispersive x-ray fluorescence spectrophotometer (ED-XRF) X-supreme 8000 from 5 g polished rice samples. The published protocol of ED-XRF was used to estimate both the micro-nutrients [11]. These genotypes were grouped into three different phenotypic classes based on milled rice grain iron content for our study as high (> 4 mg kg^− 1^), medium (3–4 mg kg^− 1^) and low (< 3 mg kg^− 1^) and for zinc content as high (> 20 mg kg^− 1^), medium (15–20 mg kg^− 1^) and low (< 15 mg kg^− 1^). Principal component analysis (PCA) and scatter plots were generated as per standard procedure following previous publications [39, 40, 77].

### DNA isolation, PCR amplification and visualization of markers for Fe and Zn content in polished rice

The seedlings of the 102 genotypes were grown and the leaf samples were collected from 20 days old seedling for genomic DNA isolation. The DNA was isolated following standard protocol [78]. One hundred SSR and gene specific markers across the genome were used for the study (Additional file 4: Table S2). The gene specific markers were selected based on reported literature on Fe-Zn content in rice. PCR was performed in 20 μl reaction volume and amplified by following our earlier report [17]. Agarose gel (3.5%) in TBE buffer (pH 8.0) was used for electrophoresis. Ethidium Bromide stain was used for visualisation of the amplicons. 50 bp DNA ladder was used to determine the size of the amplicon.

### Analyses for determination of population structure, genetic diversity parameters and association of markers

A matrix was constructed on the basis of presence and/or absence of alleles for each genotype-primer combination. PowerMarker Ver3.25 software was used for estimating allele number, polymorphic information content (PIC), allele frequency, gene diversity and heterozygosis [79]. NEI coefficient for dissimilarity index was calculated and unweighted neighbor joining un-rooted tree was constructed [80] with bootstrap value of 1000 by using DARwin5 program [81]. A Bayesian clustering approach was followed for estimation of genetic structure by taking probable sub-populations (K) and higher delta K-value using the STRUCTURE 2.3.6 software [82]. A 150,000 burn-in period with 150,000 MCMC replications and K value run of 10 times was used for estimating population structure. The highest value of delta K estimated from Evanno table was taken as number of probable sub-populations present in the model and used in next step to detect the sub-population values in the panel population [17, 39, 40, 83, 84]. The deviation from Hardy-Weinberg expectation within and between the population structures (F_IT,_ F_IS,_ F_ST_) was estimated through Analysis of molecular variance (AMOVA) using GenAlEx 6.5 software [85]. Marker-trait association for Fe and Zn content of polished rice was performed using TASSEL 5 program [86].

Linkage disequilibrium plot was constructed following earlier reports [17]. In order to enhance accuracy of marker- trait association, false discovery rate (FDR) and adjusted *p* values (*q* values) were calculated using the methods described in previous studies [40].

### Gene expression analysis by quantitative real time-PCR

Two genotypes Kalanmak (high grain Fe and Zn) and Swarna (low grain Fe and Zn) were used for studying expression level of two genes *OsZIP6* and *OsZIP8* under Fe-Zn normal and deficient condition. Eleven day old seedlings were exposed to Fe-Zn normal and deficient condition under hydroponics culture in Yoshida growth medium was used for control, whereas both Fe and Zn were omitted from Yoshida medium for deficiency condition [72, 73, 87]. Expression analysis in response to Fe-Zn deficiency was performed for *OsZIP6* and *OsZIP8* genes using real-time primers [72]. Prior to real-time expression analysis, the total mRNA isolation, yield and purity evaluation of RNA were performed following the methods of earlier reports [88]. The increase/decrease of expression (fold change) of the *OsZIP6* and *OsZIP8* genes under Fe-Zn deficiency condition as compared to controlled condition was calculated by using 2^-ΔΔ Ct^ method [89], where normalization was performed with rice β-tubulin gene [88]. Three technical replicates each from three independent biological replicates were taken used for the study following earlier reports [88].

## Supplementary information

**Additional file 1: Figure S1.** Scree plot generated showing four component traits and eigen values generated by using 102 rice genotypes.

**Additional file 2: Figure S2.** The distribution pattern of F_ST_ values (A) in the two sub-populations at K = 2 (B) three sub-populations at K = 3 showing a symmetric shape.

**Additional file 3: Table S1.** Days to 50% flowering, grain Fe content, Zn content, panicles/m^2^ and grain yield of 102 genotypes including biofortified lines and check varieties studied during wet season, 2016 and 2017.

**Additional file 4: Table S2.** Information on the selected 100 molecular markers used for Zn and Fe content in *indica* rice.

**Additional file 5: Table S3.** Association of marker alleles with Fe, Zn content, panicle number and grain yield/plot in rice detected both in GLM analyses in a shortlisted panel population of 102 genotypes.

**Additional file 6: Table S4.** Association of marker alleles with Fe, Zn content, panicle number and grain yield/plot in rice detected both in MLM analyses in a shortlisted panel population of 102 genotypes.

## Data Availability

All data generated or analysed during this study are included in this article.

## Abbreviations

AMOVA: Analysis of molecular variance
CTAB: Cetyl Trimethyl Ammonium Bromide
FDR: False discovery rate
F_IS_: Fixation index within a population
F_IT_: Fixation index in the whole population
F_ST_: Fixation index between subpopulations
GLM: Generalized Linear Model
LD: Linkage disequilibrium
MCMC: Markov Chain Monte Carlo
MLM: Mixed Linear Model
PCA: Principal component analysis
PIC: Polymorphic information content
QTL: Quantitative trait loci
