# Supplementary figures and images for: Linkage disequilibrium mapping for grain Fe and Zn enhancing QTLs useful for nutrient dense rice breeding
Source: BMC Plant Biol. 2020 Feb 4;20:57. doi: 10.1186/s12870-020-2262-4 (PMC7001215; doi:10.1186/s12870-020-2262-4)

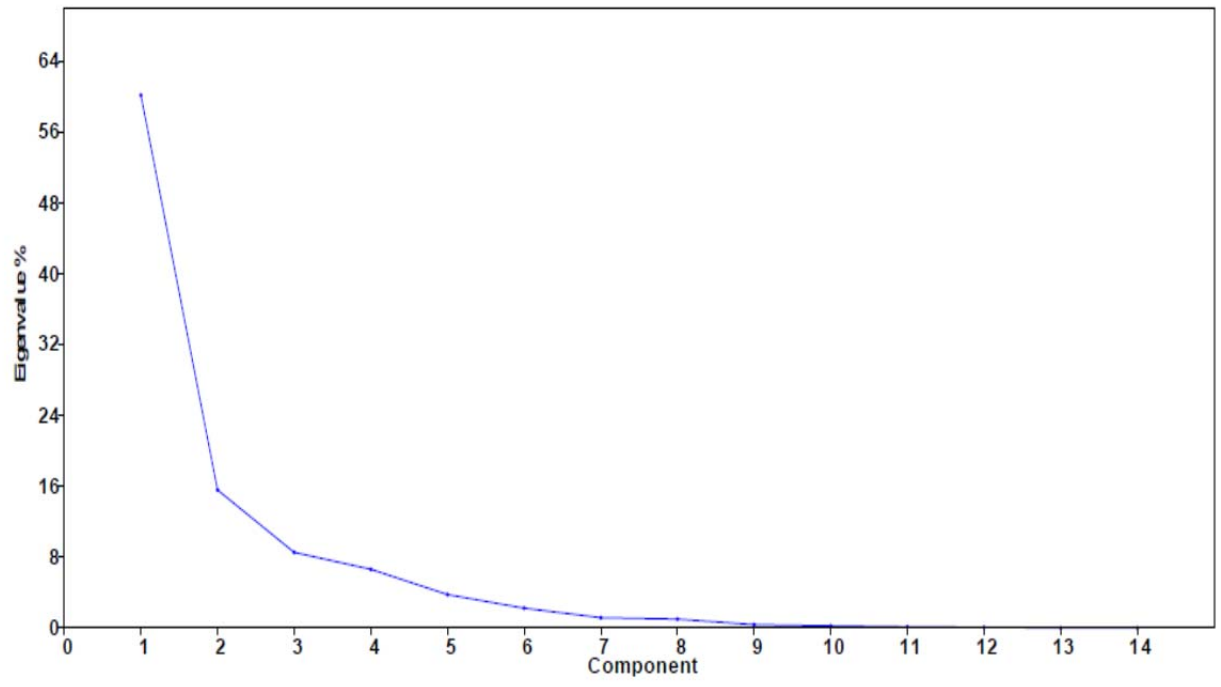

Supplement: Supplementary file 1 — Additional file 1: Figure S1. Scree plot generated showing four component traits and eigen values generated by using 102 rice genotypes. [file 12870_2020_2262_MOESM1_ESM.pdf]

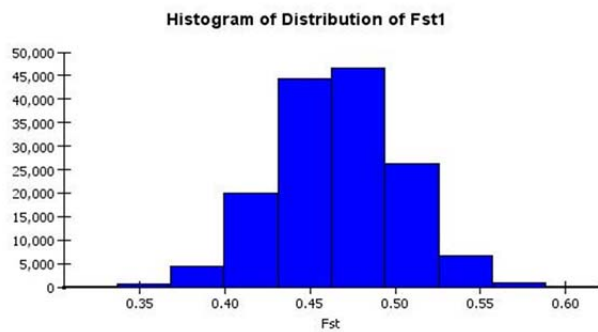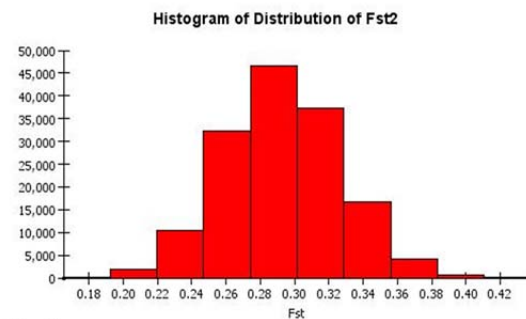

**K=2**

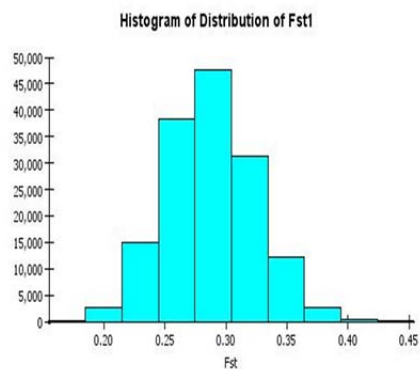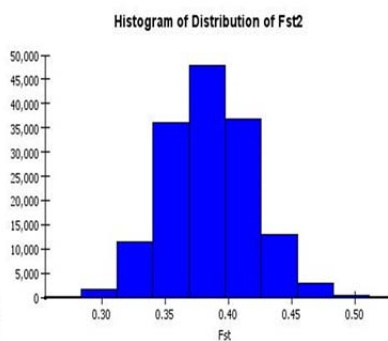

**K=3**

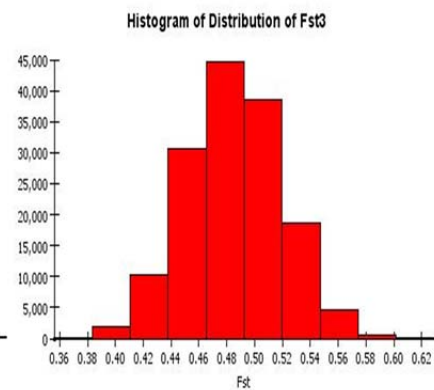

Supplement: Supplementary file 2 — Additional file 2: Figure S2. The distribution pattern of FST values (A) in the two sub-populations at K = 2 (B) three sub-populations at K = 3 showing a symmetric shape. [file 12870_2020_2262_MOESM2_ESM.pdf]
