# Supplementary material for: Linkage disequilibrium mapping for grain Fe and Zn enhancing QTLs useful for nutrient dense rice breeding
Source: BMC Plant Biol. 2020 Feb 4;20:57. doi: 10.1186/s12870-020-2262-4 (PMC7001215; doi:10.1186/s12870-020-2262-4)
Supplement: Supplementary file 3 — Additional file 3: Table S1. Days to 50% flowering, grain Fe content, Zn content, panicles/m2 and grain yield of 102 genotypes including biofortified lines and check varieties studied during wet season, 2016 and 2017. [file 12870_2020_2262_MOESM3_ESM.docx]

Additional file 3: **Table S1.** Days to 50% flowering, grain Fe content, Zn content, panicles/m^2^ and grain yield of 102 genotypes including biofortified lines and check varieties studied during wet season, 2016 and 2017

| Sl. No. | National testing No./Accession No./Name of the genotype | Days to 50% flowering | | Iron content in ppm | | Zinc content in ppm | | Panicles/m^2^ | | Grain yield  (kg/ha) | |
| --- | --- | --- | --- | --- | --- | --- | --- | --- | --- | --- | --- |
|  |  | 2016 | 2017 | 2016 | 2017 | 2016 | 2017 | 2016 | 2017 | 2016 | 2017 |
| 1 | IET23829 | 96 | 100 | 3.9 | 4.5 | 17.98 | 19.02 | 298 | 252 | 3.67 | 3.93 |
| 2 | IR64 | 90 | 94 | 3.89 | 4.11 | 17.32 | 15.72 | 271 | 301 | 4.4 | 3.82 |
| 3 | IET23834 | 92 | 86 | 3.67 | 3.33 | 19.56 | 17.04 | 286 | 248 | 3.74 | 3.38 |
| 4 | Kalanamak | 104 | 106 | 4.78 | 4.22 | 26.38 | 24.82 | 276 | 246 | 2.68 | 2.82 |
| 5 | IET23824 | 88 | 86 | 3.65 | 3.35 | 21.99 | 19.79 | 285 | 249 | 3.99 | 3.83 |
| 6 | IET23832 | 98 | 94 | 3.94 | 4.46 | 18.01 | 17.99 | 242 | 282 | 3.95 | 4.55 |
| 7 | Chittimuthyalu | 113 | 109 | 4.45 | 4.75 | 22.19 | 20.89 | 250 | 294 | 3.68 | 4.02 |
| 8 | IET24780 | 92 | 98 | 2.94 | 3.26 | 14.38 | 13.82 | 278 | 322 | 4.58 | 4.12 |
| 9 | BPT5204 | 112 | 108 | 4.4 | 4.9 | 17.35 | 15.05 | 297 | 257 | 4.61 | 4.53 |
| 10 | IET24771 | 101 | 107 | 4.23 | 3.75 | 16.16 | 14.76 | 259 | 299 | 4.91 | 5.33 |
| 11 | IET24766 | 96 | 90 | 3.43 | 3.17 | 16.67 | 13.33 | 272 | 304 | 3.72 | 4.26 |
| 12 | IET24316 | 85 | 89 | 4.25 | 4.75 | 18.34 | 16.74 | 274 | 230 | 3.34 | 4.02 |
| 13 | IET24391 | 112 | 110 | 2.68 | 3.28 | 21.28 | 19.62 | 285 | 233 | 4.26 | 3.64 |
| 14 | IET24777 | 96 | 94 | 2.75 | 2.55 | 15.53 | 12.47 | 252 | 318 | 4.53 | 5.23 |
| 15 | IET24760 | 110 | 112 | 4.35 | 3.87 | 18.24 | 19.36 | 331 | 279 | 5.32 | 4.6 |
| 16 | IET24775 | 109 | 107 | 4.36 | 4.04 | 17.52 | 18.12 | 295 | 265 | 4.71 | 5.09 |
| 17 | IET24783 | 95 | 91 | 3.26 | 3.18 | 15.31 | 17.77 | 251 | 291 | 4.17 | 4.85 |
| 18 | IET24544 | 94 | 100 | 2.01 | 2.39 | 16.39 | 17.01 | 259 | 299 | 4.21 | 5.03 |
| 19 | IET24336 | 93 | 95 | 2.76 | 3.02 | 17.36 | 16.84 | 287 | 257 | 4.68 | 4.36 |
| 20 | IET24772 | 97 | 93 | 3.78 | 3.42 | 17.24 | 15.84 | 266 | 302 | 4.02 | 4.62 |
| 21 | IET24557 | 110 | 102 | 3.55 | 4.05 | 17.54 | 18.34 | 314 | 266 | 3.53 | 4.23 |
| 22 | IET24779 | 105 | 101 | 3.91 | 3.65 | 21.34 | 19.74 | 299 | 263 | 4.26 | 3.7 |
| 23 | IET24774 | 103 | 107 | 2.92 | 3.06 | 17.39 | 15.01 | 265 | 319 | 3.86 | 4.82 |
| 24 | IET24787 | 92 | 96 | 4.13 | 3.67 | 18.02 | 16.68 | 262 | 302 | 3.32 | 2.98 |
| 25 | IET25441 | 98 | 102 | 3.28 | 3.72 | 17.36 | 14.68 | 284 | 240 | 4.52 | 4.48 |
| 26 | IET25443 | 110 | 106 | 3.29 | 3.83 | 11.89 | 9.19 | 312 | 264 | 3.67 | 2.87 |
| 27 | IET25444 | 112 | 108 | 3.06 | 3.34 | 16.75 | 14.19 | 278 | 232 | 4.56 | 4.94 |
| 28 | IET25445 | 104 | 106 | 3.43 | 3.17 | 15.38 | 12.66 | 273 | 307 | 4.69 | 5.09 |
| 29 | IET25446 | 94 | 92 | 3.81 | 4.15 | 22.74 | 19.86 | 263 | 297 | 3.81 | 4.11 |
| 30 | IET25447 | 105 | 103 | 3.72 | 3.88 | 14.68 | 15.26 | 265 | 299 | 5.34 | 4.8 |
| 31 | IET25449 | 97 | 93 | 3.42 | 2.86 | 14.24 | 17.04 | 269 | 243 | 4.61 | 4.03 |
| 32 | IET25450 | 100 | 96 | 4.77 | 4.23 | 21.16 | 23.92 | 271 | 321 | 3.01 | 3.41 |
| 33 | IET25452 | 107 | 103 | 4.2 | 3.6 | 15.37 | 18.03 | 257 | 301 | 4.54 | 4.36 |
| 34 | IET25453 | 93 | 87 | 2.66 | 3.32 | 16.67 | 13.33 | 262 | 302 | 3.95 | 4.57 |
| 35 | IET25454 | 85 | 91 | 3.95 | 3.65 | 20.21 | 18.79 | 242 | 180 | 2.98 | 2.46 |
| 36 | IET25457 | 97 | 99 | 2.54 | 2.86 | 15.57 | 18.23 | 236 | 292 | 3.31 | 4.05 |
| 37 | IET25459 | 100 | 102 | 2.79 | 3.01 | 14.33 | 16.97 | 274 | 308 | 4.1 | 5.04 |
| 38 | IET25460 | 106 | 102 | 3.46 | 3.74 | 14.57 | 11.97 | 236 | 276 | 5.21 | 5.49 |
| 39 | IET25461 | 95 | 93 | 3.27 | 3.73 | 22.21 | 18.43 | 295 | 231 | 3.64 | 3.04 |
| 40 | IET25463 | 91 | 99 | 3.49 | 2.91 | 14.64 | 17.26 | 274 | 322 | 5.18 | 5.54 |
| 41 | IET25464 | 89 | 93 | 2.91 | 2.69 | 14.17 | 17.05 | 288 | 234 | 3.16 | 3.84 |
| 42 | IET25465 | 102 | 98 | 4.35 | 4.05 | 20.14 | 18.94 | 258 | 236 | 3.46 | 3.94 |
| 43 | Gontra Bidhan3 | 107 | 103 | 2.97 | 2.49 | 13.15 | 9.75 | 279 | 259 | 3.74 | 4.24 |
| 44 | IET25469 | 93 | 95 | 3.35 | 2.93 | 19.87 | 16.33 | 238 | 268 | 3.39 | 3.73 |
| 45 | IET25470 | 96 | 90 | 2.99 | 2.79 | 19.67 | 16.33 | 267 | 233 | 4.87 | 4.27 |
| 46 | IET25471 | 103 | 99 | 3 | 3.44 | 17.94 | 14.06 | 267 | 295 | 4.52 | 4.7 |
| 47 | DRRH3 | 97 | 99 | 2.58 | 2.78 | 11.01 | 13.89 | 259 | 277 | 4.92 | 5.56 |
| 48 | IET25472 | 88 | 94 | 3.57 | 3.81 | 27.97 | 23.03 | 254 | 254 | 3.27 | 4.03 |
| 49 | IET25473 | 99 | 105 | 3.12 | 3.78 | 16.57 | 17.39 | 315 | 287 | 4.93 | 5.05 |
| 50 | IET25474 | 106 | 102 | 3.08 | 3.5 | 16.12 | 18.88 | 268 | 320 | 4.51 | 3.918 |
| 51 | IET25475 | 97 | 91 | 3.54 | 3.82 | 23.67 | 20.51 | 268 | 228 | 3.33 | 4.01 |
| 52 | IET25477 | 101 | 99 | 3.41 | 2.83 | 25.39 | 22.61 | 282 | 318 | 3.97 | 3.77 |
| 53 | IET25478 | 103 | 105 | 2.59 | 3.15 | 17.32 | 15.28 | 309 | 279 | 3.21 | 5.81 |
| 54 | IET25479 | 106 | 104 | 3.61 | 4.13 | 16.94 | 13.48 | 310 | 310 | 4.26 | 4.28 |
| 55 | Lalmeeta | 117 | 113 | 2.42 | 2.88 | 11.75 | 9.15 | 299 | 277 | 2.98 | 3.26 |
| 56 | Abhimanyu | 117 | 123 | 3.6 | 4.1 | 9.57 | 12.07 | 264 | 236 | 3.69 | 4.33 |
| 57 | Kalobhutia | 121 | 123 | 3.71 | 4.09 | 12.73 | 10.17 | 268 | 244 | 3.31 | 3.77 |
| 58 | Sadakajam | 119 | 113 | 4.72 | 4.3 | 16.9 | 14.1 | 245 | 221 | 3.56 | 3.66 |
| 59 | Geetanjali | 133 | 129 | 3.32 | 3.7 | 12.98 | 10.62 | 279 | 251 | 4.21 | 4.37 |
| 60 | Kakhru | 124 | 128 | 2.51 | 3.05 | 14.84 | 11.16 | 287 | 267 | 3.39 | 4.15 |
| 61 | Boanti | 119 | 123 | 3.01 | 3.43 | 13.36 | 14.16 | 259 | 277 | 3.11 | 3.77 |
| 62 | Tulsimukul | 123 | 119 | 3.95 | 3.75 | 12.21 | 14.79 | 221 | 179 | 2.39 | 3.39 |
| 63 | Kokilpatri | 112 | 108 | 2.24 | 3.06 | 9.37 | 12.17 | 265 | 205 | 3.57 | 3.01 |
| 64 | Basmatikarnal | 122 | 126 | 2.94 | 3.56 | 9.54 | 12.2 | 251 | 315 | 3.43 | 2.87 |
| 65 | Kalonunia | 131 | 135 | 4.19 | 3.69 | 14.65 | 11.95 | 223 | 267 | 2.76 | 3.22 |
| 66 | SafedLuchai2 | 108 | 114 | 3.22 | 3.86 | 14.38 | 17.36 | 284 | 242 | 2.78 | 2.96 |
| 67 | Bankra | 132 | 138 | 2.64 | 2.48 | 13.56 | 11.14 | 241 | 199 | 3.52 | 3.78 |
| 68 | Moongi | 123 | 127 | 3.17 | 2.65 | 8.37 | 10.77 | 247 | 183 | 2.57 | 3.17 |
| 69 | Swarnakranti | 124 | 126 | 3.74 | 3.3 | 11.34 | 9.76 | 321 | 279 | 3.55 | 4.13 |
| 70 | Kalojeera | 98 | 102 | 3.68 | 3.4 | 8.57 | 7.43 | 134 | 180 | 2.59 | 3.19 |
| 71 | Ketekijoha | 111 | 115 | 2.75 | 2.15 | 18.69 | 16.11 | 248 | 272 | 3.44 | 4.98 |
| 72 | Maudamani | 108 | 104 | 1.64 | 1.92 | 14.58 | 15.26 | 229 | 261 | 7.25 | 7.55 |
| 73 | Tarori Basmati | 114 | 110 | 1.7 | 2.3 | 15.36 | 12.76 | 246 | 274 | 2.14 | 2.82 |
| 74 | Mamihunger | 113 | 109 | 2.67 | 2.41 | 18.28 | 20.72 | 154 | 170 | 2.84 | 2.24 |
| 75 | Sneha | 113 | 109 | 2.54 | 1.88 | 21.39 | 18.91 | 255 | 211 | 3.01 | 3.41 |
| 76 | Savitri | 121 | 123 | 2.41 | 2.21 | 10.36 | 12.78 | 272 | 244 | 5.32 | 5.22 |
| 77 | CR Dhan 101 | 97 | 95 | 2.19 | 1.63 | 14.32 | 11.72 | 274 | 230 | 4.09 | 4.69 |
| 78 | CR Dhan 907 | 110 | 112 | 2.41 | 1.59 | 15.65 | 13.05 | 288 | 248 | 3.58 | 3.98 |
| 79 | CR Dhan 801 | 112 | 114 | 1.87 | 2.01 | 16.35 | 13.65 | 302 | 268 | 5.67 | 5.47 |
| 80 | Chinikamini | 114 | 116 | 2.15 | 1.89 | 13.85 | 11.25 | 264 | 234 | 4.32 | 3.9 |
| 81 | Nuakalajeera | 108 | 112 | 2.74 | 2.94 | 17.65 | 14.35 | 256 | 218 | 3.32 | 3.34 |
| 82 | Moti | 107 | 113 | 1.86 | 2.12 | 16.84 | 14.24 | 259 | 217 | 4.51 | 3.69 |
| 83 | Nuadhusura | 110 | 114 | 2.64 | 3.16 | 17.65 | 16.75 | 238 | 202 | 2.49 | 3.51 |
| 84 | Heera | 74 | 70 | 1.79 | 1.89 | 17.68 | 15.4 | 231 | 189 | 3.26 | 3.74 |
| 85 | Jalmagna | 130 | 132 | 2.57 | 2.37 | 16.46 | 13.56 | 277 | 247 | 3.86 | 2.62 |
| 86 | AC44756 | 111 | 113 | 2.41 | 2.67 | 14.24 | 13.84 | 221 | 177 | 2.22 | 1.58 |
| 87 | AC44755 | 114 | 112 | 1.98 | 2.46 | 16.12 | 14.68 | 178 | 134 | 2.98 | 2.76 |
| 88 | AC44754 | 112 | 116 | 2.61 | 2.29 | 15.74 | 14.26 | 192 | 168 | 2.36 | 2.04 |
| 89 | AC44753 | 113 | 111 | 2.03 | 2.25 | 13.38 | 15.78 | 236 | 188 | 2.54 | 1.66 |
| 90 | Swarna-Sub 1 | 115 | 111 | 1.79 | 2.09 | 13.64 | 15.96 | 325 | 275 | 6.09 | 5.31 |
| 91 | Ranjit | 123 | 127 | 1.85 | 2.05 | 15.89 | 14.09 | 295 | 263 | 5.73 | 4.47 |
| 92 | Swarna | 120 | 124 | 2.61 | 1.49 | 15.09 | 12.91 | 302 | 348 | 6.31 | 5.49 |
| 93 | Jaya | 108 | 110 | 1.88 | 2.28 | 17.48 | 15.12 | 261 | 319 | 4.57 | 3.85 |
| 94 | Samalei | 114 | 108 | 1.99 | 2.61 | 15.84 | 11.2 | 234 | 270 | 4.44 | 3.76 |
| 95 | AC44752 | 108 | 110 | 1.33 | 1.63 | 17.68 | 15.32 | 195 | 141 | 2.37 | 1.63 |
| 96 | Lalat | 103 | 97 | 1.25 | 1.47 | 20.79 | 18.51 | 254 | 282 | 4.18 | 4.46 |
| 97 | MTU1010 | 98 | 92 | 1.57 | 1.37 | 16.94 | 13.94 | 264 | 294 | 4.51 | 4.81 |
| 98 | Naveen | 102 | 98 | 2.26 | 1.74 | 14.94 | 12.14 | 271 | 309 | 5.82 | 5.18 |
| 99 | Satabdi | 93 | 87 | 1.67 | 1.83 | 17.85 | 15.65 | 255 | 215 | 4.01 | 3.99 |
| 100 | Pooja | 123 | 127 | 1.78 | 2.18 | 14.95 | 11.75 | 333 | 287 | 5.37 | 6.53 |
| 101 | Sarala | 128 | 132 | 2.14 | 1.64 | 16.9 | 13.5 | 324 | 272 | 5.85 | 5.31 |
| 102 | Agnisar | 100 | 104 | 1.12 | 1.78 | 14.54 | 11.46 | 234 | 186 | 2.18 | 3.38 |
|  | LSD_5%_ | 5.85 | 5.45 | 0.61 | 0.596 | 3.11 | 3.44 | 55.97 | 53.6 | 0.90 | 0.94 |
|  | CV% | 2.8 | 2.6 | 10.2 | 9.9 | 9.9 | 10.4 | 10.8 | 10.6 | 11.4 | 11.5 |
