# Supplementary material for: Linkage disequilibrium mapping for grain Fe and Zn enhancing QTLs useful for nutrient dense rice breeding
Source: BMC Plant Biol. 2020 Feb 4;20:57. doi: 10.1186/s12870-020-2262-4 (PMC7001215; doi:10.1186/s12870-020-2262-4)
Supplement: Supplementary file 4 — Additional file 4: Table S2. Information on the selected 100 molecular markers used for Zn and Fe content in indica rice. [file 12870_2020_2262_MOESM4_ESM.docx]

Additional file 4: **Table S2.** Information on the selected 100 molecular markers used for Zn and Fe content in *indica* rice

| Sl.  No. | Marker Name | Chr.  No. | Sequence forward | Sequence reverse | Repeat motif | Expected  band size (bp) | Reference |
| --- | --- | --- | --- | --- | --- | --- | --- |
| 1 | RM243 | 1 | GATCTGCAGACTGCAGTTGC | AGCTGCAACGATGTTGTCC | CT18 | 116 | Anuradha et al. 2012 |
| 2 | RM488 | 1 | CAGCTAGGGTTTTGAGGCTG | TAGCAACAACCAGCGTATGC | GA17 | 177 | Anuradha et al. 2012 |
| 3 | RM490 | 1 | ATCTGCACACTGCAAACACC | AGCAAGCAGTGCTTTCAGAG | CT13 | 101 | Anuradha et al. 2012 |
| 4 | RM574 | 5 | GGCGAATTCTTTGCACTTGG | ACGGTTTGGTAGGGTGTCAC | GA11 | 155 | Anuradha et al. 2012 |
| 5 | RM122 | 5 | GAGTCGATGTAATGTCATCAGTGC | GAAGGAGGTATCGCTTTGTTGGAC | (GA)7A(GA)2A(GA)11 | 227 | Anuradha et al. 2012 |
| 6 | RM234 | 7 | ACAGTATCCAAGGCCCTGG | CACGTGAGACAAAGACGGAG | CT25 | 156 | Anuradha et al. 2012 |
| 7 | RM248 | 7 | TCCTTGTGAAATCTGGTCCC | GTAGCCTAGCATGGTGCATG | CT25 | 102 | Anuradha et al. 2012 |
| 8 | RM8007 | 7 | AATAGGATGGATCATGGATA | CATCTCATCAGGAACCTAAC | AT40 | 178 | Anuradha et al. 2012 |
| 9 | RM17 | 12 | TGCCCTGTTATTTTCTTCTCTC | GGTGATCCTTTCCCATTTCA | GA)21 | 184 | Anuradha et al. 2012 |
| 10 | RM260 | 12 | ACTCCACTATGACCCAGAG | GAACAATCCCTTCTACGATCG | (CT)34 | 111 | Anuradha et al. 2012 |
| 11 | RM7 | 3 | TTCGCCATGAAGTCTCTCG | CCTCCCATCATTTCGTTGTT | (GA)19 | 180 | Anuradha et al. 2012 |
| 12 | RM517 | 3 | GGCTTACTGGCTTCGATTTG | CGTCTCCTTTGGTTAGTGCC | (CT)15 | 266 | Anuradha et al. 2012 |
| 13 | RM501 | 7 | GCCCAATTAATGTACAGGCG | ATATCGTTTAGCCGTGCTGC | (TC)10(TA)21 | 179 | Anuradha et al. 2012 |
| 14 | OSZIP4 | 8 | GCGAAAGCAACAGTGATCATGGCGACTTTC | GCAGCTCTTGGTTGCTCTGAAGATCTCATG | CT18 | 116 | Anuradha et al. 2012 |
| 15 | RM594 | 1 | GCCACCAGTAAAAGCAATAC | TTGATCTGCTAGTGAGACCC |  |  | Anuradha et al. 2012 |
| 16 | RM3412 | 1 | AAAGCAGGTTTTCCTCCTCC | CCCATGTGCAATGTGTCTTC | (GA)n | 300 | Anuradha et al. 2012 |
| 17 | RM5638 | 1 | GGCTTCCTCATCGCCATC | CTGAGCAGCATTCCAGTCTG | (CT)17 | 211 | Anuradha et al. 2012 |
| 18 | RM6712 | 3 | GCGCATCATCACTTCATCAG | AGATGAGCCTATCAGCTGCC | (AAG)13 | 203 | Anuradha et al. 2012 |
| 19 | RM168 | 3 | TGCTGCTTGCCTGCTTCCTTT | GAAACGAATCAATCCACGGC | (TAT)8 | 97 | Anuradha et al. 2012 |
| 20 | RM5626 | 3 | GCAGACGAGATGAGATCG | GTAGAGGATGGGCAGCAG | T15(GT)14 | 116 | Anuradha et al. 2012 |
| 21 | RM3392 | 3 | GTCCAATGATTCGTTCCCAC | CTTCACCGTTCACCAATTCC | (AAG)11 | 188 | Anuradha et al. 2012 |
| 22 | RM1278 | 3 | ATATAAAGGTGGCACGACAG | GCACTTGAACTCTAATTCTCC | (CT)17 | 164 | Anuradha et al. 2012 |
| 23 | RM471 | 4 | ACGCACAAGCAGATGATGAG | GGGAGAAGACGAATGTTTGC | (AG)17 | 131 | Anuradha et al. 2012 |
| 24 | RM521 | 2 | TTCCCTTATTCCTGCTCTCC | GGGATTTGCAGTGAGCTAGC | (GA)12 | 106 | Anuradha et al. 2012 |
| 25 | RM6209 | 5 | GCTTCGTCTTCCTCATCTCG | ACTTGTTGACTGCTCCCTCG | (TC)14 | 260 | Ishimaru et al |
| 26 | RM80 | 8 | TTGAAGGCGCTGAAGGAG | CATCAACCTCGTCTTCACCG | (CGG)8 | 77 | Anuradha et al. 2012 |
| 27 | OSZIP8 | 7 | GGCGACGTGTTCTTCCTCGT | TCGTCGGTGAGGTTGTCGAA | (CCG)10 |  |  |
| 28 | RM152 | 8 | AAGGAGAAGTTCTTCGCCCAGTGC | GCCCATTAGTGACTGCTCCTAGTCG | (GGC)10 | 151 | Akagi etal.1996 |
| 29 | RM440 | 5 | CATGCAACAACGTCACCTTC | ATGGTTGGTAGGCACCAAAG | (CTT)22 | 169 |  |
| 30 | RM432 | 7 | TTCTGTCTCACGCTGGATTG | AGCTGCGTACGTGATGAATG | (CATC)9 | 187 |  |
| 31 | RM434 | 9 | GCCTCATCCCTCTAACCCTC | CAAGAAAGATCAGTGCGTGG | (TC)12 | 152 |  |
| 32 | RM 3 | 6 | ACACTGTAGCGGCCACTG | CCTCCACTGCTCCACATCTT | (GA)2GG(GA)25 | 145 | LongXu jian.et al 2015 |
| 33 | RM 1 | 1 | GCGAAAACACAATGCAAAAA | GCGTTGGTTGGACCTGAC | (GA)26 | 113 | Brar et al. 2015 |
| 34 | RM 144 | 11 | TGCCCTGGCGCAAATTTGATCC | GCTAGAGGAGATCAGATGGTAGTGCATG | (ATT)11 | 237 | Brar et al. 2015 |
| 35 | RM 201 | 9 | CTCGTTTATTACCTACAGTACC | CTACCTCCTTTCTAGACCGATA | (CT)17 | 158 | Brar et al. 2015 |
| 36 | RM 205 | 9 | CTGGTTCTGTATGGGAGCAG | CTGGCCCTTCACGTTTCAGTG | (CT)25 | 122 | Brar et al. 2015 |
| 37 | RM 270 | 6 | GGCCGTTGGTTCTAAAATC | TGCGCAGTATCATCGGCGAG | (GA)13 | 108 | Brar et al. 2015 |
| 38 | RM 335 | 4 | GTACACACCCACATCGAGAAG | GCTCTATGCGAGTATCCATGG | (CTT)25 | 104 | Brar et al. 2015 |
| 39 | RM154 | 2 | GACGGTGACGCACTTTATGAACC | CGATCTGCGAGAAACCCTCTCC | (GA)21 | 183 | LongXu jian.et al 2015 |
| 40 | RM211 | 2 | CCGATCTCATCAACCAACTG | CTTCACGAGGATCTCAAAGG | (TC)3A(TC)18 | 161 | LongXu jian.et al 2015 |
| 41 | RM202 | 11 | CAGATTGGAGATGAAGTCCTCC | CCAGCAAGCATGTCAATGTA | (CT)30 | 189 | LongXu jian.et al 2015 |
| 42 | RM293 | 3 | TCGTTGGGAGGTATGGTACC | CTTTATCTGATCCTTGGGAAGG | (GT)20 | 207 | LongXu jian.et al 2015 |
| 43 | RM85 | 3 | CCAAAGATGAAACCTGGATTG | GCACAAGGTGAGCAGTCC | (TGG)5(TCT)12 | 107 | LongXu jian.et al 2015 |
| 44 | RM407 | 8 | GATTGAGGAGACGAGCCATC | CTTTTTCAGATCTGCGCTCC | (AG)13 | 172 | LongXu jian.et al 2015 |
| 45 | RM237 | 1 | CAAATCCCGACTGCTGTCC | TGGGAAGAGAGCACTACAGC | (CT)18 | 130 | LongXu jian.et al 2015 |
| 46 | RM259 | 1 | TGGAGTTTGAGAGGAGGG | CTTGTTGCATGGTGCCATGT | (CT)17 | 162 | LongXu jian.et al 2015 |
| 47 | RM421 | 5 | AGCTCAGGTGAAACATCCAC | ATCCAGAATCCATTGACCCC | (AGAT)6 | 234 | LongXu jian.et al 2015 |
| 48 | RM235 | 12 | AGAAGCTAGGGCTAACGAAC | TCACCTGGTCAGCCTCTTTC | (CT)24 | 124 | LongXu jian.et al 2015 |
| 49 | RM1337 | 12 | GTGCAATGCTGAGGAGTATC | CTGAGAATCTGGAGTGCTTG | (AG)21 | 210 | LongXu jian.et al 2015 |
| 50 | RM3409 | 8 | AGTGCCTCCTCCGTTCTCC | AGTGCTACGGGTGCTAGCTC | (CT)17 | 96 | LongXu jian.et al 2015 |
| 51 | RM105 | 9 | GTCGTCGACCCATCGGAGCCAC | TGGTCGAGGTGGGGATCGGGTC | (CCT)6 | 134 | LongXu jian.et al 2015 |
| 52 | RM309 | 12 | GTAGATCACGCACCTTTCTGG | AGAAGGCCTCCGGTGAAG | (GT)13 | 169 | LongXu jian.et al 2015 |
| 53 | RM452 | 2 | CTGATCGAGAGCGTTAAGGG | GGGATCAAACCACGTTTCTG | (GTC)9 | 209 | LongXu jian.et al 2015 |
| 54 | RM204 | 6 | GTGACTGACTTGGTCATAGGG | GCTAGCCATGCTCTCGTACC | (CT)44 | 169 | LongXu jian.et al 2015 |
| 55 | RM137 | 8 | GACATCGCCACCAGCCCACCAC | CGGGTGGTCCCCGAGGATCTTG | (CT)7 | 218 | LongXu jian.et al 2015 |
| 56 | RM1789 | 8 | GGAAATGTACAGATGTGTGG | CAATCTCGCAATTTTTCATA | (AT)16 | 125 | LongXu jian.et al 2015 |
| 57 | RM6641 | 2 | GGGTCTCGATTCTCAGTTGG | CAGAACCACTCATGCACACC | (GTA)14 | 160 | Garcia-Oliveira et al. 2008 |
| 58 | RM296 | 9 | CACATGGCACCAACCTCC | GCCAAGTCATTCACTACTCTGG | (GA)10 | 123 | Garcia-Oliveira et al. 2008 |
| 59 | RM3331 | 12 | CCTCCTCCATGAGCTAATGC | AGGAGGAGCGGATTTCTCTC | (CT)15 | 129 | Garcia-Oliveira et al. 2008 |
| 60 | RM31 | 5 | GATCACGATCCACTGGAGCT | AAGTCCATTACTCTCCTCCC | (GA)15 | 140 |  |
| 61 | RM429 | 7 | TCCCTCCAGCAATGTCTTTC | CCTTCATCTTGCTTTCCACC | (TG)10 | 159 |  |
| 62 | RM556 | 8 | ACTCCAAACCTCACTGCACC | TAGCACACTGAACAGCTGGC | (CCAG)6 | 93 |  |
| 63 | RM585 | 6 | CAGTCTTGCTCCGTTTGTTG | CTGTGACTGACTTGGTCATAGG | (TC)45 | 233 |  |
| 64 | RM23 | 1 | CATTGGAGTGGAGGCTGG | GTCAGGCTTCTGCCATTCTC | (GA)15 | 145 |  |
| 65 | RM34 | 9 | GAAATGGCAATGTGTGCG | GCCGGAGAACCCTAGCTC | (CT)17(TC)2 | 161 | Brar et al. 2015 |
| 66 | RM53 |  | ACGTCTCGACGCATCAATGG | CACAAGAACTTCCTCGGTAC | (GA)14 | 182 |  |
| 67 | RM300 | 2 | GCTTAAGGACTTCTGCGAACC | CAACAGCGATCCACATCATC | (GTT)14 | 121 | Brar et al. 2015 |
| 68 | RM315 | 1 | GAGGTACTTCCTCCGTTTCAC | AGTCAGCTCACTGTGCAGTG | (AT)4(GT)10 | 133 | Brar et al. 2015 |
| 69 | RM339 | 8 | GTAATCGATGCTGTGGGAAG | GAGTCATGTGATAGCCGATATG | (CTT)8CCT(CTT)5 | 148 | Brar et al. 2015 |
| 70 | RM400 | 6 | ACACCAGGCTACCCAAACTC | CGGAGAGATCTGACATGTGG | (ATA)63 | 321 | Brar et al. 2015 |
| 71 | RM528 | 6 | GGCATCCAATTTTACCCCTC | AAATGGAGCATGGAGGTCAC | (AGAT)9 | 232 | Brar et al. 2015 |
| 72 | RM486 | 1 | CCCCCCTCTCTCTCTCTCTC | TAGCCACATCAACAGCTTGC | (CT)14 | 104 | LongXu jian.et al 2015 |
| 73 | RM340 | 6 | GGTAAATGGACAATCCTATGGC | GACAAATATAAGGGCAGTGTGC | (CTT)8T3(CTT)14 | 163 | LongXu jian.et al 2015 |
| 74 | RM1132 | 7 | ATCACCTGAGAAACATCCGG | CTCCTCCCACGTCAAGGTC | (AG)12 | 93 | LongXu jian.et al 2015 |
| 75 | RM441 | 11 | ACACCAGAGAGAGAGAGAGAGAG | TCTGCAACGGCTGATAGATG | (AG)13 | 189 | LongXu jian.et al 2015 |
| 76 | RM590 | 10 | CATCTCCGCTCTCCATGC | GGAGTTGGGGTCTTGTTCG | (TCT)10 | 137 | Garcia-Oliveira et al. 2008 |
| 77 | RM258 | 10 | CATCTCCGCTCTCCATGC | GGAGTTGGGGTCTTGTTCG | (GA)21(GGA)3 | 148 | Garcia-Oliveira et al. 2008 |
| 78 | GRMM9-1 | 9 | TCGGATTCTCGTGGTACA | GAAGTTGGACTGGTAGGACA |  | 275 | Chandel et al. 2011 |
| 79 | GRMM9-2 | 9 | AACCGGGTTTCTTACCTG | CCCAAGCTGCTAAACAGT |  | 153 | Chandel et al. 2011 |
| 80 | OsNAC | 3 | AGCGAGAAGCAAGCAAGAAG | ATGCCCTGGATATCGTCGTA |  | 600 | Gande et al. 2014 |
| 81 | OsZIP8A | 7 | ATGAGGACGAACACCACCAC | CGGAGGGAGGGAGTAGTAATG |  | 880 | Gande et al. 2014 |
| 82 | OsZIP8C | 7 | TGTAAAGAGGGAAAAGGGAGCTA | GGCGAGTACATTCACTTCCATT |  | 927 | Gande et al. 2014 |
| 83 | OsYSL4E | 5 | TATGCATGCGGTGGATGA | GTACTTTGGGAGGCCCTTCA |  | 851 | Soman et al. 2014 |
| 84 | OsMTP1A | 5 | TCTCTCCTCCCCATCTCCAA | GCTGGTTACAGCGAAAGCAC |  | 961 | Soman et al. 2014 |
| 85 | OsNRAMP5G | 7 | GATCATTACGTATGTCGTTGTATCTC | AATTACAAGGATACATGAGCCACCT |  | 992 | Soman et al. 2014 |
| 86 | IRMM9-1 | 9 | GTGACGAGCGAGCGGATG | TGTTCAACAGATTCTTCTTCG |  | 205 | Soman et al. 2014 |
| 87 | OsYSL1 | 4 | GTGATGACAAGAACCACAACGG | AGCTGGTGGAAGATGCGG |  | 230 | Suma 2015 |
| 88 | OsYSL2A | 2 | CCGAGATAGAGAGGTGAGAC | ATGATGATGACGGTGTAGACGA |  | 154 | Suma 2015 |
| 89 | OsYSL2B | 2 | GGATTCGTCTACACCGTCATC | GTACGTCTTCCTGTTCAGAGCC |  | 285 | Suma 2015 |
| 90 | OsYSL5 | 4 | CAAATCCACCGGAGATGC | GATGACCACGCACAGCAG |  | 227 | Suma 2015 |
| 91 | OsYSL6 | 4 | CGGACGATGCGGAGATCA | CGTGATGAGGCAGAACAGCAC |  | 167 | Suma 2015 |
| 92 | OsYSL11 | 4 | CCAACAACAGTAGCTCGGC | GTGGTCAGGGACAGCTTCAT |  | 259 | Suma 2015 |
| 93 | OsZIP6A | 5 | GGCCTCTTCTCCCTCGTC | ACTTTCTTGGTGGTGGGGATT |  | 166 | Suma 2015 |
| 94 | OsZIP6B | 5 | GCACTCAGACGACACGGAC | AGTGGAACACGATCCCAATCT |  | 215 | Suma 2015 |
| 95 | OsZIP7 | 5 | CCAGGTTCTACGAGGGCAAG | CTGTGTGAGTGCGAGTGAGGT |  | 172 | Suma 2015 |
| 96 | OsZIP8 | 7 | ACAACCTCACCGACGACTG | CACCTGTCCCTCGTGCTC |  | 266 | Suma 2015 |
| 97 | OsNRAMP1A | 7 | GGTACAGGATTTGCTTTCAACC | ACCACAACCTCCAGTTTCCTTA |  | 128 | Suma 2015 |
| 98 | OsNRAMP1B | 7 | CCTTTGAGCTACCATTTGCTCT | GTTTATCCCGATGATGACGAAC |  | 134 | Suma 2015 |
| 99 | OsFER1 | 11 | CTTTCCGCCATGCTTCCT | CTGAGCACCTCCTTCCCTTT |  | 167 | Suma 2015 |
| 100 | OsFER2 | 12 | CGCCTCCTCTCACTTAATCC | CTGAGCACCTCCTTCCCTTT |  | 194 | Suma 2015 |
