# Supplementary material for: Linkage disequilibrium mapping for grain Fe and Zn enhancing QTLs useful for nutrient dense rice breeding
Source: BMC Plant Biol. 2020 Feb 4;20:57. doi: 10.1186/s12870-020-2262-4 (PMC7001215; doi:10.1186/s12870-020-2262-4)
Supplement: Supplementary file 5 — Additional file 5: Table S3. Association of marker alleles with Fe, Zn content, panicle number and grain yield/plot in rice detected both in GLM analyses in a shortlisted panel population of 102 genotypes. [file 12870_2020_2262_MOESM5_ESM.docx]

Additional file 5: **Table S3**. Association of marker alleles with Fe , Zn content, panicle number and grain yield/plot in rice detected both in GLM analyses in a shortlisted panel population of 102 genotypes

| Trait | Marker | F value | P value | R^2^ |  |  |  |  |  |  |  |  |  |  |  |  |  |
| --- | --- | --- | --- | --- | --- | --- | --- | --- | --- | --- | --- | --- | --- | --- | --- | --- | --- |
| Iron | RM243 | 6.21777 | 0.01429 | 0.05854 |  |  |  |  |  |  |  |  |  |  |  |  |  |
| Iron | RM488 | 6.63259 | 0.01148 | 0.0622 |  |  |  |  |  |  |  |  |  |  |  |  |  |
| Iron | RM490 | 7.42014 | 0.00761 | 0.06908 |  |  |  |  |  |  |  |  |  |  |  |  |  |
| Iron | RM574 | 31.27305 | 1.95E-07 | 0.23823 |  |  |  |  |  |  |  |  |  |  |  |  |  |
| Iron | RM122 | 4.3147 | 0.04035 | 0.04136 |  |  |  |  |  |  |  |  |  |  |  |  |  |
| Iron | RM234 | 16.81381 | 8.41E-05 | 0.14394 |  |  |  |  |  |  |  |  |  |  |  |  |  |
| Iron | RM248 | 4.4945 | 0.03648 | 0.04301 |  |  |  |  |  |  |  |  |  |  |  |  |  |
| Iron | RM260 | 6.41551 | 0.01287 | 0.06029 |  |  |  |  |  |  |  |  |  |  |  |  |  |
| Iron | RM7 | 8.87945 | 0.00362 | 0.08155 |  |  |  |  |  |  |  |  |  |  |  |  |  |
| Iron | RM517 | 8.71483 | 0.00393 | 0.08016 |  |  |  |  |  |  |  |  |  |  |  |  |  |
| Iron | OSZIP4 | 115.1457 | 2.49E-18 | 0.5352 |  |  |  |  |  |  |  |  |  |  |  |  |  |
| Iron | RM594 | 25.11118 | 2.34E-06 | 0.20071 |  |  |  |  |  |  |  |  |  |  |  |  |  |
| Iron | RM3412 | 4.81839 | 0.03047 | 0.04597 |  |  |  |  |  |  |  |  |  |  |  |  |  |
| Iron | RM5638 | 5.07356 | 0.02648 | 0.04829 |  |  |  |  |  |  |  |  |  |  |  |  |  |
| Iron | RM6712 | 22.35779 | 7.43E-06 | 0.18272 |  |  |  |  |  |  |  |  |  |  |  |  |  |
| Iron | RM168 | 14.70112 | 2.20E-04 | 0.12817 |  |  |  |  |  |  |  |  |  |  |  |  |  |
| Iron | RM3392 | 12.63863 | 5.79E-04 | 0.11221 |  |  |  |  |  |  |  |  |  |  |  |  |  |
| Iron | RM1278 | 4.99708 | 0.02761 | 0.04759 |  |  |  |  |  |  |  |  |  |  |  |  |  |
| Iron | RM521 | 10.23987 | 0.00184 | 0.09289 |  |  |  |  |  |  |  |  |  |  |  |  |  |
| Iron | RM6209 | 5.3654 | 0.02258 | 0.05092 |  |  |  |  |  |  |  |  |  |  |  |  |  |
| Iron | RM80 | 16.39877 | 1.01E-04 | 0.14088 |  |  |  |  |  |  |  |  |  |  |  |  |  |
| Iron | OSZIP8 | 156.0936 | 3.85E-22 | 0.60952 |  |  |  |  |  |  |  |  |  |  |  |  |  |
| Iron | RM152 | 5.815 | 0.01771 | 0.05495 |  |  |  |  |  |  |  |  |  |  |  |  |  |
| Iron | RM440 | 31.98448 | 1.48E-07 | 0.24234 |  |  |  |  |  |  |  |  |  |  |  |  |  |
| Iron | RM432 | 12.58771 | 5.93E-04 | 0.1118 |  |  |  |  |  |  |  |  |  |  |  |  |  |
| Iron | RM434 | 24.96748 | 2.48E-06 | 0.19979 |  |  |  |  |  |  |  |  |  |  |  |  |  |
| Iron | RM1 | 40.61016 | 5.75E-09 | 0.28881 |  |  |  |  |  |  |  |  |  |  |  |  |  |
| Iron | RM144 | 11.72587 | 8.95E-04 | 0.10495 |  |  |  |  |  |  |  |  |  |  |  |  |  |
| Iron | RM201 | 17.18095 | 7.13E-05 | 0.14662 |  |  |  |  |  |  |  |  |  |  |  |  |  |
| Iron | RM205 | 6.9176 | 0.00988 | 0.0647 |  |  |  |  |  |  |  |  |  |  |  |  |  |
| Iron | RM154 | 7.48671 | 0.00736 | 0.06965 |  |  |  |  |  |  |  |  |  |  |  |  |  |
| Iron | RM211 | 10.40718 | 0.0017 | 0.09426 |  |  |  |  |  |  |  |  |  |  |  |  |  |
| Iron | RM85 | 7.4305 | 0.00757 | 0.06917 |  |  |  |  |  |  |  |  |  |  |  |  |  |
| Iron | RM407 | 45.10353 | 1.15E-09 | 0.31084 |  |  |  |  |  |  |  |  |  |  |  |  |  |
| Iron | RM235 | 5.8958 | 0.01696 | 0.05568 |  |  |  |  |  |  |  |  |  |  |  |  |  |
| Iron | RM3409 | 13.64019 | 3.61E-04 | 0.12003 |  |  |  |  |  |  |  |  |  |  |  |  |  |
| Iron | RM309 | 16.30812 | 1.06E-04 | 0.14021 |  |  |  |  |  |  |  |  |  |  |  |  |  |
| Iron | RM204 | 9.2999 | 0.00293 | 0.08509 |  |  |  |  |  |  |  |  |  |  |  |  |  |
| Iron | RM137 | 26.70131 | 1.22E-06 | 0.21074 |  |  |  |  |  |  |  |  |  |  |  |  |  |
| Iron | RM1789 | 156.0936 | 3.85E-22 | 0.60952 |  |  |  |  |  |  |  |  |  |  |  |  |  |
| Iron | RM6641 | 8.44403 | 0.00451 | 0.07787 |  |  |  |  |  |  |  |  |  |  |  |  |  |
| Iron | RM296 | 39.30605 | 9.27E-09 | 0.28216 |  |  |  |  |  |  |  |  |  |  |  |  |  |
| Iron | RM31 | 10.62573 | 0.00152 | 0.09605 |  |  |  |  |  |  |  |  |  |  |  |  |  |
| Iron | RM429 | 8.40883 | 0.00459 | 0.07757 |  |  |  |  |  |  |  |  |  |  |  |  |  |
| Iron | RM585 | 12.98372 | 4.92E-04 | 0.11492 |  |  |  |  |  |  |  |  |  |  |  |  |  |
| Iron | RM23 | 27.61915 | 8.38E-07 | 0.21642 |  |  |  |  |  |  |  |  |  |  |  |  |  |
| Iron | RM53 | 24.46885 | 3.06E-06 | 0.19659 |  |  |  |  |  |  |  |  |  |  |  |  |  |
| Iron | RM339 | 11.69699 | 9.08E-04 | 0.10472 |  |  |  |  |  |  |  |  |  |  |  |  |  |
| Iron | RM400 | 17.4641 | 6.28E-05 | 0.14868 |  |  |  |  |  |  |  |  |  |  |  |  |  |
| Iron | RM528 | 9.39512 | 0.0028 | 0.08588 |  |  |  |  |  |  |  |  |  |  |  |  |  |
| Iron | RM340 | 20.91522 | 1.38E-05 | 0.17297 |  |  |  |  |  |  |  |  |  |  |  |  |  |
| Iron | RM1132 | 4.14297 | 0.04445 | 0.03978 |  |  |  |  |  |  |  |  |  |  |  |  |  |
| Iron | RM441 | 6.91559 | 0.00989 | 0.06468 |  |  |  |  |  |  |  |  |  |  |  |  |  |
| Iron | GRMM9-2 | 12.44353 | 6.35E-04 | 0.11066 |  |  |  |  |  |  |  |  |  |  |  |  |  |
| Iron | OSNAC | 4.02021 | 0.04766 | 0.03865 |  |  |  |  |  |  |  |  |  |  |  |  |  |
| Iron | OSZIP88 | 11.44893 | 0.00102 | 0.10273 |  |  |  |  |  |  |  |  |  |  |  |  |  |
| Iron | OSZIP8C | 16.79759 | 8.47E-05 | 0.14382 |  |  |  |  |  |  |  |  |  |  |  |  |  |
| Iron | OSYSL4E | 13.46509 | 3.92E-04 | 0.11867 |  |  |  |  |  |  |  |  |  |  |  |  |  |
| Iron | OSMTP1A | 4.15606 | 0.04412 | 0.0399 |  |  |  |  |  |  |  |  |  |  |  |  |  |
| Iron | OSNRAMP5G | 15.19635 | 1.75E-04 | 0.13192 |  |  |  |  |  |  |  |  |  |  |  |  |  |
| Iron | RMM9-1 | 6.8715 | 0.01013 | 0.0643 |  |  |  |  |  |  |  |  |  |  |  |  |  |
| Iron | OSYSL1 | 52.37911 | 9.52E-11 | 0.34374 |  |  |  |  |  |  |  |  |  |  |  |  |  |
| Iron | OSYSL5 | 30.1647 | 3.02E-07 | 0.23174 |  |  |  |  |  |  |  |  |  |  |  |  |  |
| Iron | OSYSL6 | 27.00044 | 1.08E-06 | 0.2126 |  |  |  |  |  |  |  |  |  |  |  |  |  |
| Iron | OSYSL11 | 17.14587 | 7.24E-05 | 0.14636 |  |  |  |  |  |  |  |  |  |  |  |  |  |
| Iron | OSZIP6A | 5.86773 | 0.01722 | 0.05543 |  |  |  |  |  |  |  |  |  |  |  |  |  |
| Iron | OSZIP6B | 71.85256 | 2.12E-13 | 0.41811 |  |  |  |  |  |  |  |  |  |  |  |  |  |
| Iron | OSZIP8 | 30.77777 | 2.37E-07 | 0.23534 |  |  |  |  |  |  |  |  |  |  |  |  |  |
| Iron | OSNRAMP1A | 26.30947 | 1.43E-06 | 0.20829 |  |  |  |  |  |  |  |  |  |  |  |  |  |
| Iron | OSNRAMP1B | 101.0401 | 7.62E-17 | 0.50259 |  |  |  |  |  |  |  |  |  |  |  |  |  |
| Iron | OSFER1 | 20.9887 | 1.34E-05 | 0.17348 |  |  |  |  |  |  |  |  |  |  |  |  |  |
| Zinc | RM243 | 6.99294 | 0.0095 | 0.06536 |  |  |  |  |  |  |  |  |  |  |  |  |  |
| Zinc | RM260 | 5.99267 | 0.01611 | 0.05654 |  |  |  |  |  |  |  |  |  |  |  |  |  |
| Zinc | RM517 | 4.095 | 0.04568 | 0.03934 |  |  |  |  |  |  |  |  |  |  |  |  |  |
| Zinc | RM80 | 11.86398 | 8.38E-04 | 0.10606 |  |  |  |  |  |  |  |  |  |  |  |  |  |
| Zinc | RM1 | 4.51418 | 0.03608 | 0.04319 |  |  |  |  |  |  |  |  |  |  |  |  |  |
| Zinc | RM211 | 4.72029 | 0.03217 | 0.04508 |  |  |  |  |  |  |  |  |  |  |  |  |  |
| Zinc | RM202 | 4.9456 | 0.02841 | 0.04713 |  |  |  |  |  |  |  |  |  |  |  |  |  |
| Zinc | RM235 | 10.8583 | 0.00136 | 0.09795 |  |  |  |  |  |  |  |  |  |  |  |  |  |
| Zinc | RM296 | 6.71905 | 0.01097 | 0.06296 |  |  |  |  |  |  |  |  |  |  |  |  |  |
| Zinc | RM585 | 4.42005 | 0.03803 | 0.04233 |  |  |  |  |  |  |  |  |  |  |  |  |  |
| Zinc | RM300 | 11.98471 | 7.91E-04 | 0.10702 |  |  |  |  |  |  |  |  |  |  |  |  |  |
| Zinc | RM339 | 5.32614 | 0.02307 | 0.05057 |  |  |  |  |  |  |  |  |  |  |  |  |  |
| Zinc | RM340 | 5.59171 | 0.01998 | 0.05296 |  |  |  |  |  |  |  |  |  |  |  |  |  |
| Zinc | RM1132 | 9.0313 | 0.00335 | 0.08283 |  |  |  |  |  |  |  |  |  |  |  |  |  |
| Zinc | GRMM9-1 | 13.00392 | 4.87E-04 | 0.11507 |  |  |  |  |  |  |  |  |  |  |  |  |  |
| Zinc | OSNAC | 8.51071 | 0.00436 | 0.07843 |  |  |  |  |  |  |  |  |  |  |  |  |  |
| Zinc | OSYSL4E | 6.70008 | 0.01108 | 0.06279 |  |  |  |  |  |  |  |  |  |  |  |  |  |
| Zinc | OSZIP6A | 5.14049 | 0.02553 | 0.04889 |  |  |  |  |  |  |  |  |  |  |  |  |  |
| Zinc | OSFER1 | 4.07584 | 0.04618 | 0.03916 |  |  |  |  |  |  |  |  |  |  |  |  |  |
| PN | RM243 | 16.28077 | 1.07E-04 | 0.14001 |  |  |  |  |  |  |  |  |  |  |  |  |  |
| PN | RM488 | 5.30997 | 0.02327 | 0.05042 |  |  |  |  |  |  |  |  |  |  |  |  |  |
| PN | RM574 | 5.23432 | 0.02425 | 0.04974 |  |  |  |  |  |  |  |  |  |  |  |  |  |
| PN | RM248 | 18.23846 | 4.45E-05 | 0.15425 |  |  |  |  |  |  |  |  |  |  |  |  |  |
| PN | RM17 | 14.62367 | 2.28E-04 | 0.12758 |  |  |  |  |  |  |  |  |  |  |  |  |  |
| PN | RM7 | 14.01318 | 3.03E-04 | 0.12291 |  |  |  |  |  |  |  |  |  |  |  |  |  |
| PN | OSZIP4 | 17.3904 | 6.49E-05 | 0.14814 |  |  |  |  |  |  |  |  |  |  |  |  |  |
| PN | RM594 | 8.61885 | 0.00413 | 0.07935 |  |  |  |  |  |  |  |  |  |  |  |  |  |
| PN | RM3392 | 9.9083 | 0.00217 | 0.09015 |  |  |  |  |  |  |  |  |  |  |  |  |  |
| PN | RM6209 | 5.62756 | 0.01959 | 0.05328 |  |  |  |  |  |  |  |  |  |  |  |  |  |
| PN | OSZIP8 | 18.68005 | 3.66E-05 | 0.1574 |  |  |  |  |  |  |  |  |  |  |  |  |  |
| PN | RM440 | 13.87657 | 3.23E-04 | 0.12186 |  |  |  |  |  |  |  |  |  |  |  |  |  |
| PN | RM432 | 9.11102 | 0.00322 | 0.0835 |  |  |  |  |  |  |  |  |  |  |  |  |  |
| PN | RM1 | 19.3224 | 2.76E-05 | 0.16193 |  |  |  |  |  |  |  |  |  |  |  |  |  |
| PN | RM201 | 8.2573 | 0.00496 | 0.07627 |  |  |  |  |  |  |  |  |  |  |  |  |  |
| PN | RM85 | 12.94277 | 5.01E-04 | 0.1146 |  |  |  |  |  |  |  |  |  |  |  |  |  |
| PN | RM407 | 8.31255 | 0.00482 | 0.07675 |  |  |  |  |  |  |  |  |  |  |  |  |  |
| PN | RM421 | 7.28154 | 0.00818 | 0.06787 |  |  |  |  |  |  |  |  |  |  |  |  |  |
| PN | RM235 | 6.04008 | 0.01571 | 0.05696 |  |  |  |  |  |  |  |  |  |  |  |  |  |
| PN | RM3409 | 4.90213 | 0.02909 | 0.04673 |  |  |  |  |  |  |  |  |  |  |  |  |  |
| PN | RM1789 | 18.68005 | 3.66E-05 | 0.1574 |  |  |  |  |  |  |  |  |  |  |  |  |  |
| PN | RM296 | 10.69384 | 0.00148 | 0.09661 |  |  |  |  |  |  |  |  |  |  |  |  |  |
| PN | RM31 | 4.76448 | 0.03139 | 0.04548 |  |  |  |  |  |  |  |  |  |  |  |  |  |
| PN | RM556 | 18.72712 | 3.58E-05 | 0.15773 |  |  |  |  |  |  |  |  |  |  |  |  |  |
| PN | RM585 | 7.80515 | 0.00624 | 0.0724 |  |  |  |  |  |  |  |  |  |  |  |  |  |
| PN | RM23 | 15.80571 | 1.33E-04 | 0.13648 |  |  |  |  |  |  |  |  |  |  |  |  |  |
| PN | RM34 | 6.86821 | 0.01014 | 0.06427 |  |  |  |  |  |  |  |  |  |  |  |  |  |
| PN | RM53 | 10.79912 | 0.0014 | 0.09747 |  |  |  |  |  |  |  |  |  |  |  |  |  |
| PN | RM339 | 8.26377 | 0.00494 | 0.07633 |  |  |  |  |  |  |  |  |  |  |  |  |  |
| PN | RM400 | 4.5259 | 0.03584 | 0.0433 |  |  |  |  |  |  |  |  |  |  |  |  |  |
| PN | RM340 | 12.95157 | 4.99E-04 | 0.11466 |  |  |  |  |  |  |  |  |  |  |  |  |  |
| PN | RM1132 | 7.17598 | 0.00864 | 0.06696 |  |  |  |  |  |  |  |  |  |  |  |  |  |
| PN | OSZIP8C | 4.39191 | 0.03864 | 0.04207 |  |  |  |  |  |  |  |  |  |  |  |  |  |
| PN | OSNRAMP5G | 4.61641 | 0.03408 | 0.04413 |  |  |  |  |  |  |  |  |  |  |  |  |  |
| PN | IRMM9-1 | 7.0437 | 0.00926 | 0.0658 |  |  |  |  |  |  |  |  |  |  |  |  |  |
| PN | OSYSL1 | 13.16177 | 4.52E-04 | 0.11631 |  |  |  |  |  |  |  |  |  |  |  |  |  |
| PN | OSYSL5 | 5.10181 | 0.02607 | 0.04854 |  |  |  |  |  |  |  |  |  |  |  |  |  |
| PN | OSYSL6 | 4.95425 | 0.02827 | 0.0472 |  |  |  |  |  |  |  |  |  |  |  |  |  |
| PN | OSZIP6A | 6.57862 | 0.01181 | 0.06173 |  |  |  |  |  |  |  |  |  |  |  |  |  |
| PN | OSZIP6B | 7.05685 | 0.00919 | 0.06592 |  |  |  |  |  |  |  |  |  |  |  |  |  |
| PN | OSZIP8 | 5.44381 | 0.02164 | 0.05163 |  |  |  |  |  |  |  |  |  |  |  |  |  |
| PN | OSNRAMP1A | 26.69468 | 1.22E-06 | 0.2107 |  |  |  |  |  |  |  |  |  |  |  |  |  |
| PN | OSNRAMP1B | 13.24436 | 4.35E-04 | 0.11695 |  |  |  |  |  |  |  |  |  |  |  |  |  |
| PN | OSFER1 | 25.83769 | 1.73E-06 | 0.20533 |  |  |  |  |  |  |  |  |  |  |  |  |  |
| Yield | RM243 | 4.05979 | 0.0466 | 0.03901 |  |  |  |  |  |  |  |  |  |  |  |  |  |
| Yield | RM488 | 6.42518 | 0.0128 | 0.06037 |  |  |  |  |  |  |  |  |  |  |  |  |  |
| Yield | RM490 | 4.65633 | 0.03333 | 0.04449 |  |  |  |  |  |  |  |  |  |  |  |  |  |
| Yield | RM234 | 8.02211 | 0.00559 | 0.07426 |  |  |  |  |  |  |  |  |  |  |  |  |  |
| Yield | RM248 | 5.92531 | 0.0167 | 0.05594 |  |  |  |  |  |  |  |  |  |  |  |  |  |
| Yield | RM17 | 11.91911 | 8.16E-04 | 0.1065 |  |  |  |  |  |  |  |  |  |  |  |  |  |
| Yield | RM517 | 12.86597 | 5.20E-04 | 0.11399 |  |  |  |  |  |  |  |  |  |  |  |  |  |
| Yield | RM6712 | 7.65217 | 0.00676 | 0.07108 |  |  |  |  |  |  |  |  |  |  |  |  |  |
| Yield | RM168 | 5.32582 | 0.02307 | 0.05057 |  |  |  |  |  |  |  |  |  |  |  |  |  |
| Yield | RM3392 | 4.20854 | 0.04284 | 0.04039 |  |  |  |  |  |  |  |  |  |  |  |  |  |
| Yield | RM1278 | 4.28698 | 0.04098 | 0.04111 |  |  |  |  |  |  |  |  |  |  |  |  |  |
| Yield | RM521 | 6.65615 | 0.01133 | 0.06241 |  |  |  |  |  |  |  |  |  |  |  |  |  |
| Yield | RM440 | 4.75041 | 0.03164 | 0.04535 |  |  |  |  |  |  |  |  |  |  |  |  |  |
| Yield | RM434 | 4.53931 | 0.03558 | 0.04342 |  |  |  |  |  |  |  |  |  |  |  |  |  |
| Yield | RM3 | 6.83165 | 0.01034 | 0.06395 |  |  |  |  |  |  |  |  |  |  |  |  |  |
| Yield | RM201 | 4.71011 | 0.03235 | 0.04498 |  |  |  |  |  |  |  |  |  |  |  |  |  |
| Yield | RM154 | 8.15715 | 0.00522 | 0.07542 |  |  |  |  |  |  |  |  |  |  |  |  |  |
| Yield | RM259 | 6.15235 | 0.01479 | 0.05796 |  |  |  |  |  |  |  |  |  |  |  |  |  |
| Yield | RM421 | 6.89217 | 0.01002 | 0.06448 |  |  |  |  |  |  |  |  |  |  |  |  |  |
| Yield | RM585 | 4.55401 | 0.03529 | 0.04356 |  |  |  |  |  |  |  |  |  |  |  |  |  |
| Yield | RM34 | 6.06991 | 0.01546 | 0.05723 |  |  |  |  |  |  |  |  |  |  |  |  |  |
| Yield | RM300 | 8.81349 | 0.00374 | 0.081 |  |  |  |  |  |  |  |  |  |  |  |  |  |
| Yield | RM339 | 11.30774 | 0.00109 | 0.10159 |  |  |  |  |  |  |  |  |  |  |  |  |  |
| Yield | RM486 | 4.37264 | 0.03905 | 0.04189 |  |  |  |  |  |  |  |  |  |  |  |  |  |
| Yield | RM1132 | 11.952 | 8.03E-04 | 0.10676 |  |  |  |  |  |  |  |  |  |  |  |  |  |
