# Supplementary material for: Linkage disequilibrium mapping for grain Fe and Zn enhancing QTLs useful for nutrient dense rice breeding
Source: BMC Plant Biol. 2020 Feb 4;20:57. doi: 10.1186/s12870-020-2262-4 (PMC7001215; doi:10.1186/s12870-020-2262-4)
Supplement: Supplementary file 6 — Additional file 6: Table S4. Association of marker alleles with Fe, Zn content, panicle number and grain yield/plot in rice detected both in MLM analyses in a shortlisted panel population of 102 genotypes. [file 12870_2020_2262_MOESM6_ESM.docx]

Additional file 6: **Table S4**. Association of marker alleles with Fe , Zn content, panicle number and grain yield/plot in rice detected both in MLM analyses in a shortlisted panel population of 102 genotypes

| Trait | Marker | F value | P value | R^2^ |  |  |  |  |  |  |  |  |  |  |  |  |  |  |
| --- | --- | --- | --- | --- | --- | --- | --- | --- | --- | --- | --- | --- | --- | --- | --- | --- | --- | --- |
| Iron | RM243 | 5.85605 | 0.01733 | 0.05798 |  |  |  |  |  |  |  |  |  |  |  |  |  |  |
| Iron | RM122 | 7.11419 | 0.00892 | 0.07044 |  |  |  |  |  |  |  |  |  |  |  |  |  |  |
| Iron | RM234 | 8.05498 | 0.0055 | 0.07975 |  |  |  |  |  |  |  |  |  |  |  |  |  |  |
| Iron | RM7 | 4.05058 | 0.04684 | 0.0401 |  |  |  |  |  |  |  |  |  |  |  |  |  |  |
| Iron | RM168 | 4.93231 | 0.02862 | 0.04883 |  |  |  |  |  |  |  |  |  |  |  |  |  |  |
| Iron | RM80 | 7.60915 | 0.00691 | 0.07534 |  |  |  |  |  |  |  |  |  |  |  |  |  |  |
| Iron | RM315 | 8.22925 | 0.00503 | 0.08148 |  |  |  |  |  |  |  |  |  |  |  |  |  |  |
| Iron | RM339 | 5.24734 | 0.02408 | 0.05195 |  |  |  |  |  |  |  |  |  |  |  |  |  |  |
| Iron | RM1132 | 5.61267 | 0.01975 | 0.05557 |  |  |  |  |  |  |  |  |  |  |  |  |  |  |
| Iron | GRMM9-1 | 9.32304 | 0.0029 | 0.09231 |  |  |  |  |  |  |  |  |  |  |  |  |  |  |
| Iron | OSZIP8A | 5.2546 | 0.02398 | 0.05203 |  |  |  |  |  |  |  |  |  |  |  |  |  |  |
| Iron | OSYSL2A | 9.63229 | 0.00249 | 0.09537 |  |  |  |  |  |  |  |  |  |  |  |  |  |  |
| Iron | OSZIP6A | 7.37666 | 0.00779 | 0.07304 |  |  |  |  |  |  |  |  |  |  |  |  |  |  |
| Zinc | RM260 | 4.6087 | 0.03423 | 0.04563 |  |  |  |  |  |  |  |  |  |  |  |  |  |  |
| Zinc | RM501 | 3.96083 | 0.0493 | 0.03922 |  |  |  |  |  |  |  |  |  |  |  |  |  |  |
| Zinc | RM80 | 5.28924 | 0.02354 | 0.05237 |  |  |  |  |  |  |  |  |  |  |  |  |  |  |
| Zinc | RM434 | 4.17882 | 0.04356 | 0.04137 |  |  |  |  |  |  |  |  |  |  |  |  |  |  |
| Zinc | RM144 | 4.9201 | 0.02881 | 0.04871 |  |  |  |  |  |  |  |  |  |  |  |  |  |  |
| Zinc | RM259 | 4.59845 | 0.03442 | 0.04553 |  |  |  |  |  |  |  |  |  |  |  |  |  |  |
| Zinc | RM23 | 4.42601 | 0.0379 | 0.04382 |  |  |  |  |  |  |  |  |  |  |  |  |  |  |
| Zinc | RM300 | 7.74249 | 0.00645 | 0.07666 |  |  |  |  |  |  |  |  |  |  |  |  |  |  |
| Zinc | RM339 | 4.1887 | 0.04332 | 0.04147 |  |  |  |  |  |  |  |  |  |  |  |  |  |  |
| Zinc | RM340 | 4.78072 | 0.03111 | 0.04733 |  |  |  |  |  |  |  |  |  |  |  |  |  |  |
| Zinc | RM1132 | 6.3645 | 0.01322 | 0.06301 |  |  |  |  |  |  |  |  |  |  |  |  |  |  |
| Zinc | GRMM9-1 | 12.41426 | 6.44E-04 | 0.12291 |  |  |  |  |  |  |  |  |  |  |  |  |  |  |
| Zinc | OSYSL1 | 4.13992 | 0.04453 | 0.04099 |  |  |  |  |  |  |  |  |  |  |  |  |  |  |
| PN | RM248 | 5.4383 | 0.0217 | 0.05384 |  |  |  |  |  |  |  |  |  |  |  |  |  |  |
| PN | RM17 | 11.26493 | 0.00112 | 0.11153 |  |  |  |  |  |  |  |  |  |  |  |  |  |  |
| PN | RM517 | 7.79023 | 0.00629 | 0.07713 |  |  |  |  |  |  |  |  |  |  |  |  |  |  |
| PN | RM3392 | 4.57817 | 0.03482 | 0.04533 |  |  |  |  |  |  |  |  |  |  |  |  |  |  |
| PN | RM440 | 4.78325 | 0.03107 | 0.04736 |  |  |  |  |  |  |  |  |  |  |  |  |  |  |
| PN | RM85 | 3.98538 | 0.04862 | 0.03946 |  |  |  |  |  |  |  |  |  |  |  |  |  |  |
| PN | RM421 | 11.14884 | 0.00118 | 0.11038 |  |  |  |  |  |  |  |  |  |  |  |  |  |  |
| PN | RM31 | 4.77291 | 0.03125 | 0.04726 |  |  |  |  |  |  |  |  |  |  |  |  |  |  |
| PN | RM556 | 4.90142 | 0.02911 | 0.04853 |  |  |  |  |  |  |  |  |  |  |  |  |  |  |
| PN | RM23 | 4.12242 | 0.04497 | 0.04082 |  |  |  |  |  |  |  |  |  |  |  |  |  |  |
| PN | RM340 | 5.74925 | 0.01835 | 0.05692 |  |  |  |  |  |  |  |  |  |  |  |  |  |  |
| PN | OSNRAMP1A | 11.87069 | 8.35E-04 | 0.11753 |  |  |  |  |  |  |  |  |  |  |  |  |  |  |
| PN | OSFER1 | 9.85209 | 0.00223 | 0.09755 |  |  |  |  |  |  |  |  |  |  |  |  |  |  |
| Yield | RM243 | 4.3019 | 0.04064 | 0.04259 |  |  |  |  |  |  |  |  |  |  |  |  |  |  |
| Yield | RM248 | 5.01002 | 0.02742 | 0.0496 |  |  |  |  |  |  |  |  |  |  |  |  |  |  |
| Yield | RM17 | 7.11104 | 0.00894 | 0.07041 |  |  |  |  |  |  |  |  |  |  |  |  |  |  |
| Yield | RM517 | 14.7466 | 2.16E-04 | 0.14601 |  |  |  |  |  |  |  |  |  |  |  |  |  |  |
| Yield | RM421 | 5.55647 | 0.02036 | 0.05501 |  |  |  |  |  |  |  |  |  |  |  |  |  |  |
| Yield | RM452 | 4.13473 | 0.04466 | 0.04094 |  |  |  |  |  |  |  |  |  |  |  |  |  |  |
| Yield | RM23 | 6.55352 | 0.01196 | 0.06489 |  |  |  |  |  |  |  |  |  |  |  |  |  |  |
| Yield | RM34 | 5.16896 | 0.02513 | 0.05118 |  |  |  |  |  |  |  |  |  |  |  |  |  |  |
| Yield | RM339 | 4.47416 | 0.0369 | 0.0443 |  |  |  |  |  |  |  |  |  |  |  |  |  |  |
| Yield | RM1132 | 4.78822 | 0.03098 | 0.04741 |  |  |  |  |  |  |  |  |  |  |  |  |  |  |
| Yield | IRMM9-1 | 7.51206 | 0.00726 | 0.07438 |  |  |  |  |  |  |  |  |  |  |  |  |  |  |
| Yield | OSFER1 | 5.00146 | 0.02755 | 0.04952 |  |  |  |  |  |  |  |  |  |  |  |  |  |  |
